# Efficient microbial colony growth dynamics quantification with ColTapp, an automated image analysis application

**DOI:** 10.1038/s41598-020-72979-4

**Published:** 2020-09-30

**Authors:** Julian Bär, Mathilde Boumasmoud, Roger D. Kouyos, Annelies S. Zinkernagel, Clément Vulin

**Affiliations:** 1 Department of Infectious Diseases and Hospital Epidemiology, University Hospital Zurich, University of Zurich, Zurich, Switzerland; 2 Institute of Medical Virology, University of Zurich, Zurich, Switzerland; 3 Institute of Biogeochemistry and Pollutant Dynamics, ETH Zurich, 8092 Zurich, Switzerland; 4 Department of Environmental Microbiology, 8600 Eawag, Dubendorf Switzerland

**Keywords:** Microbiology, Applied microbiology, Clinical microbiology, Environmental microbiology, Biological techniques, Software

## Abstract

Populations of genetically identical bacteria are phenotypically heterogeneous, giving rise to population functionalities that would not be possible in homogeneous populations. For instance, a proportion of non-dividing bacteria could persist through antibiotic challenges and secure population survival. This heterogeneity can be studied in complex environmental or clinical samples by spreading the bacteria on agar plates and monitoring time to growth resumption in order to infer their metabolic state distribution. We present ColTapp, the Colony Time-lapse application for bacterial colony growth quantification. Its intuitive graphical user interface allows users to analyze time-lapse images of agar plates to monitor size, color and morphology of colonies. Additionally, images at isolated timepoints can be used to estimate lag time. Using ColTapp, we analyze a dataset of *Staphylococcus aureus* time-lapse images including populations with heterogeneous lag time. Colonies on dense plates reach saturation early, leading to overestimation of lag time from isolated images. We show that this bias can be corrected by taking into account the area available to each colony on the plate. We envision that in clinical settings, improved analysis of colony growth dynamics may help treatment decisions oriented towards personalized antibiotic therapies.

## Introduction

Delayed and insufficient clearance of bacterial infections leading to treatment failure is associated with antibiotic resistance as well as antibiotic persistence. In antibiotic persistence, a subpopulation of bacteria, termed persister cells, can survive antibiotic challenges due to their phenotypic state of metabolic inactivity and then reconstitute the population by resuming growth when the stress is relieved^1^. This manifestation of phenotypic heterogeneity exists even in homogeneous liquid cultures, where each bacterial cell experiences the same local conditions^2^. On top of this, bacteria often aggregate in dense communities such as biofilms, where environmental conditions are highly heterogeneous and further promote wide phenotypic distributions^3^.

The clinical relevance of antibiotic persistence remains poorly understood. The main reason for this is that the existence and extent of persistence is difficult to assess because it is a transient phenotype and only concerns a small fraction of the bacterial population. However, these inactive bacteria are lagging longer than already actively dividing bacteria when plated on nutrient-rich agar medium, and the bacterial colonies’ macroscopic appearance time is a good proxy of single-cell lag time^4,5^. For this reason, Levin-Reisman and co-authors proposed ScanLag, a platform to monitor colony growth dynamics with time-lapse imaging^5,6^.

In clinical settings, the direct observation of colony growth dynamics from bacteria recovered from infection sites is rare^7,8^. Typically, in clinical microbiology laboratories the patient’s samples are plated and observed at a single timepoint, e.g. after 18 h of incubation. Colonies which are smaller than the bulk at that timepoint have been associated with antibiotic tolerance, persister cells and relapsing infections^9^. Small colonies can result either from mutations that affect growth rate (small colony variants^10,11^) or from heterogeneous growth resumption^5,8,12^. Reading solely the colony size at a given timepoint does not allow to distinguish between these two phenomena. Moreover, while a bimodal classification, e.g. small or “normal”, may be appropriate to describe differences in colony size due to genetic changes^13,14^, it fails to be quantitative when the range of observed colony sizes is not bimodal. This is a problem because phenotypic heterogeneity in single cell lag times typically results in unimodal colony size distributions with long tails of small colonies, and the experimenter is left with defining subjective cutoff values.

We anticipate that investigation of the antibiotic persistence phenomenon’s clinical relevance will greatly rely on colony growth dynamics analyses. Therefore, we propose to bridge the technical gap with a new analysis tool, the Colony Time-lapse application (ColTapp). Its goal is to promote wide access to colony growth dynamics quantification and enable diagnostic microbiology laboratories to further improve their routines. ColTapp is an image analysis pipeline embedded in an intuitive graphical user interface, which allows any user to derive colony sizes, growth rates and appearance times from time-lapse images of bacterial colonies. It also includes the possibility to estimate colony growth parameters from endpoint images. Additionally, it can report metrics characterizing colony color, outline and texture (morphology descriptors), and proximity of neighboring colonies (spatial metrics). We speculate that ColTapp will prove useful in a broad spectrum of microbiology applications, such as species identification in environmental samples as shown by Ernebjerg and Kishony^15^.

A technical problem common to both clinical and environmental samples is the difficulty to assess their initial bacterial density: samples can be accidentally plated at high or uneven densities. Yet, colonies compete for nutrients on the agar plates and thus the total number of colonies and their spatial distribution impacts their size at an endpoint^16^. This results in difficult appearance time estimation. To address this problem, as well as demonstrate the applicability of ColTapp, we present a dataset of *Staphylococcus aureus* bacterial colony time-lapse images and explore their response to density. The colonies’ Voronoi cell areas, one of the spatial metrics computed by ColTapp and previously explored in the context of colony growth^16^, can be taken into account to minimize the bias in appearance time estimation due to density.

## Results

### A user-friendly application implementing image- and downstream analysis

**Figure 1:**
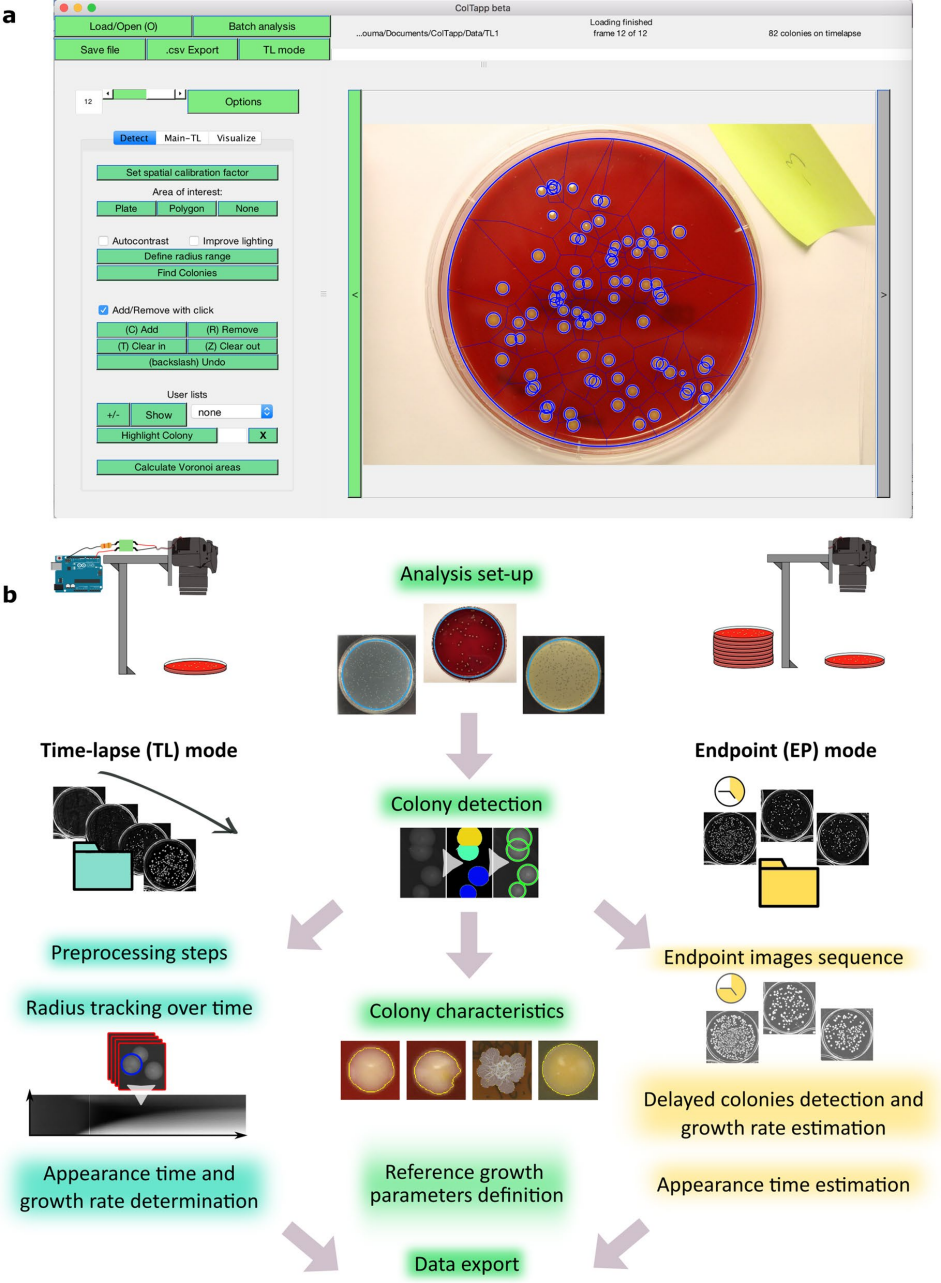
ColTapp graphical user interface and analysis workflow. (**a**) For visualization, results are overlaid onto the images: typically, the circles representing detected colonies and the edges of the Voronoi tessellation shown here in blue on the image. The main analysis panel, on the left of the image, is separated in three tabs. Here the *Detect* tab, which allows the user to detect colonies on a plate, is visualized. The *Main* tab, which allows the user to analyze either time-lapse or endpoint images and the *Visualize* tab are displayed on the Supplementary Fig. S1. (**b**) Schematic of simple image acquisition setups including a camera holder. An Arduino board (blue icon) can be used to trigger the camera automatically for time-lapse imaging of a plate (Materials and methods, Image acquisition, for implementation). ColTapp operates in two modes: either *Time-lapse* (TL) or *Endpoint* (EP) mode, depending on input data, illustrated by the two different folders (turquoise and yellow respectively). The turquoise highlighted functionalities are specific to the *Time-lapse* mode, while the yellow highlighted ones are specific to *Endpoint* mode. In the middle, the green highlighted functionalities are common to both modes. Note that each step of the workflow (apart from the two which are detailed in the following sections) has a corresponding section in the Supplementary text, which may serve as a guide to the user. For example, the “Analysis set-up” is described in the Supplementary text 2: a user may define the area of analysis on its images, as shown here in turquoise on the 3 example images. The implementation of the “colony detection” and “radius tracking over time” algorithms are described in the Materials and methods and illustrated here with small subsets of Figs. 6 and 7 respectively. The colony characteristics are illustrated here with a small subset of the Supplementary Fig. S4.

ColTapp takes images as input and implements image analysis functions to detect microbial colonies and track colony radius over time when images are part of a time-lapse sequence. Common formats (png, single page tiff, jpeg, and bmp) and either color (Red Green Blue) or grayscale images are supported. ColTapp also includes downstream analysis steps, to extract biologically relevant data such as colony growth rate and appearance time. A graphical user interface (GUI) provides access to all functionalities, displays the images and enables early visual evaluation of the results with simple data visualization options (Fig. 1a, Supplementary Fig. S1). An *Options* menu allows the user to change parameter values and access optional functionalities (Supplementary Fig. S2, Supplementary text 8) The generated data can be exported in a standard csv file (Supplementary Fig. S3, Supplementary text 9).

The application’s workflow from raw images to exported data is illustrated in Fig. 1b. The program operates in two different modes, depending on the user input data: endpoint images or time-lapse images. In the first mode, *Time-Lapse* (TL), images in a folder are considered as an ordered time-lapse image series of a single plate. In the second mode, *Endpoint* (EP), all images in a folder are considered independent from each other. Typically, they are an aggregate of images from several plated samples or from replicates of the same sample, captured at a single timepoint.

The implementation of the two main image analysis algorithms is described in the Materials and methods (Colony detection algorithm implementation and Radius tracking algorithm implementation). The user is referred to the supplementary information for more detailed directives concerning interaction with the interface, quality control functionalities, parameters optimization and performance assessment of the algorithms.

In the following sections, the downstream analyses to derive growth parameters from the resulting data are demonstrated on a dataset of 22 time-lapse movies of *S. aureus* colonies growing on blood agar plates, designed to include both homogeneous and heterogenous colony lag time and various colony densities. Note that in addition to growth parameters, a palette of colony characteristics may be exported by the user for further analysis, including spatial metrics and morphology descriptors (Supplementary Fig. S4, Supplementary text 4). Although we focus on *S. aureus* grown on blood agar in this work, ColTapp can process images of many different bacterial species growing on different colored agar media (Supplementary Fig. S5 and S6, Supplementary text 3.4).

In conclusion, an example is given to show how the ColTapp-derived measurements of radius, growth rate and appearance time can be used to assess the influence of colony density on appearance time estimation from endpoint images and how spatial metrics can be used to correct for density effects.

### Appearance time and growth rate determination from time-lapse images

**Figure 2:**
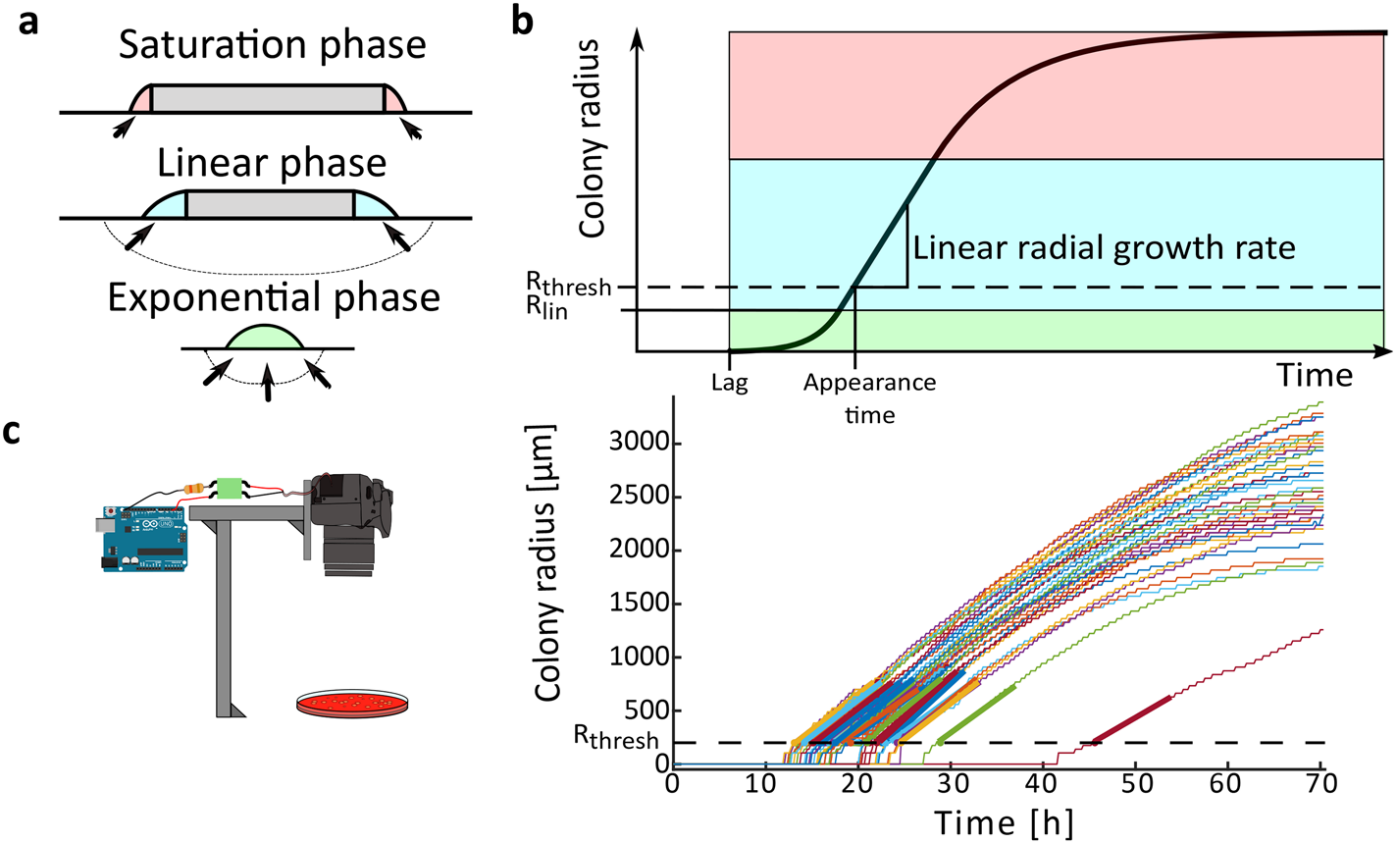
Typical colony radial growth curves. (**a**) Schematic cross section of a colony in the three growth phases. The 3 colors represent the phases (green: exponential phase, blue: linear phase, light red: saturation phase). The grey depicted areas represent bacterial cells which do not contribute to colony expansion. The arrows represent the flow of the diffusing growth substrate, steady during the linear phase and decreasing during the saturation phase. (**b**) Typical radial growth curve, with the three phases highlighted with colors corresponding to (**a**). R_lin_, displayed by the solid line, is the radius at which the radial growth rate becomes linear. R_thresh_, displayed by the dashed line, is the threshold below which a forming colony is not yet macroscopically visible. (**c**) The typical radial growth curves obtained by time-lapse image analysis in settings where the whole plate is captured (represented by the camera holder and agar plate icon). On the graph, the dashed horizontal line represents the threshold radius (R_thresh_) used for appearance time calculation and thick colored line segments represent the linear regression. Appearance time is the intersection of bold regression lines with the threshold line.

Colonies formed by non-swimming bacteria pass through three growth phases (Fig. 2a). Initially, when all bacteria forming the colony can access nutrients and thus contribute to its expansion, the colony radius increases exponentially while the colony also increases in height. Eventually, bacteria at the center of the colony do not have access to nutrients diffusing from the edge of the colony. The zone of growth at the edge of the colony becomes constant and the radial growth rate becomes linear (at this point radius = R_lin_, see Fig. 2b)^17^. Finally, when the nutrients become scarce, or when growth byproducts reach an inhibitory concentration, the colony enters the saturation phase, during which the radial growth rate starts decreasing towards 0^18^. Supplementary Fig. S7 shows the exponential phase of *S. aureus* colonies grown on sheep blood agar captured by time-lapse imaging with a macro lens. However, in typical settings where the entire plate is captured (see Materials and methods, Image acquisition), the resolution is not high enough to observe the exponential phase.

Heterogeneity of the distribution of colony appearance times can be an indication of phenotypic heterogeneity in dormancy. For this reason, appearance time is a widely used proxy for single cell lag time until first division. We define a detectable size threshold in micrometers (R_thresh_), and the time at which a colony reaches this threshold as the *appearance time* (t_app_). The minimal possible R_thresh_ depends on the image quality and needs to be set at the same value for comparisons of t_app_ in different experiments. In our analysis setting, we assume that colonies reaching this size are already in the linear growth phase (R_lin _< R_thresh_, Fig. 2b,c).

Tracking the colony radius over time using time-lapse imaging makes it possible to directly determine colony t_app_ and linear radial growth rate (GR). ColTapp determines t_app_ and GR by detecting the first of 6 consecutive frames for which a colony radius is bigger than R_thresh_ (default: 200 µm). The following radius measurements within a user-specified timespan in hours (Fr_lin_, default: 10 h) are used for a linear regression to determine GR and t_app_ (Fig. 2c). Fr_lin_ might need to be reduced to only use a timespan in which radial growth is approximately linear. Additionally, R_thresh_ can be adjusted depending on the image resolution and bacterial species investigated. The reason to perform a linear regression to determine t_app_ instead of simply observing the time when a colony radius reaches R_thresh_ is that it reduces artificial noise, to be expected if camera resolutions are low (for example if 1 pixel = 50 µm). Note that a low rate of image acquisition (long interval between time-lapse frames) might result in under-sampling of the linear growth phase, which could impact both growth rate and appearance time determination.

Growth parameters such as the duration of the exponential phase and the colony GR depend on strain characteristic properties such as replication frequency, metabolic yield^17^, cell-to-cell adhesion^19^ and surfactant production^20^. In addition, growth dynamics depend on the nutrient medium and physical properties of the agar surface such as substrate roughness^21^ or water activity^22^.

Therefore, for downstream analyses, reference growth parameters need to be measured with a control dataset (Supplementary text 6). The reference lag time is the minimal colony appearance time and can be observed by plating bacteria from a liquid culture in exponential phase which show little to no delay in growth resumption^8^. The reference linear radial growth rate is the maximal growth rate (GR_max_) observed in any experiment before competition between neighboring colonies starts to affect it.

The average colony growth rate derived from our control dataset (plates with less than 150 colonies) is 60.4 µm/h (SD = 4.7 µm/h). We define GR_max_ taking the 95th percentile (65.0 µm/h) of the growth rate distribution.

### Appearance time and growth rate estimation from endpoint images

In some laboratories, setting-up high-throughput time-lapse imaging might not be possible, thus ColTapp proposes to estimate colony lag time from endpoint images. This is possible because when bacterial cells with a lag resume growth, they rapidly reach their maximal replication rate and the resulting colonies have linear radial growth rates (GR) similar to those of colonies emerging from cells with no lag^8^. Therefore, a reference linear radial growth rate (GR) (Supplementary text 6) can be used to estimate the colony appearance time t_app_ based on colony radius R at a given timepoint (t_f_) by means of a linear extrapolation:

1 $${t}_{i}={t}_{f}-\frac{R\left({t}_{f}\right)-R({t}_{i})}{GR}$$

with R_thresh_ defining the radius (R) at time t_i_ = t_app_.

Note that using a linear extrapolation with a reference growth rate to estimate the appearance time has two strong assumptions: first, that all colonies are still in the linear phase of their growth and second, that they all have the same linear radial growth rate.

**Figure 3:**
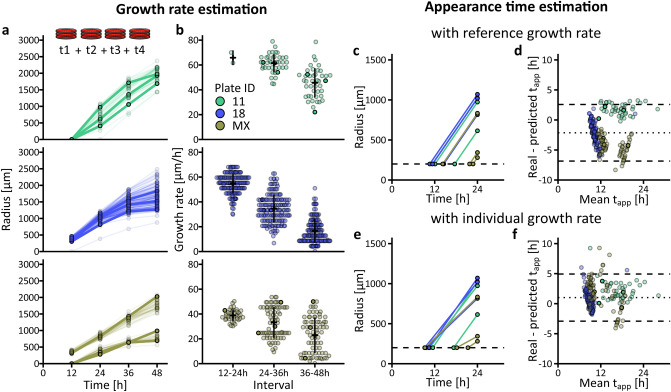
Growth rate and appearance time estimation from endpoint images. (**a**) Four images corresponding to the timepoints 12 h, 24 h, 36 h and 48 h were extracted from the full time-lapse image sequences of three independent plates and separately analyzed. Color indicates plate ID. Each dot represents radius of one colony at the given timepoint and all dots corresponding to the same colony are connected by lines (n = 44, 162 and 34 on Plate 11, 18 and MX respectively). Three colonies for each plate were highlighted. (**b**) Growth rate was estimated for each of the three intervals between the four timepoints for each colony. Crossbars indicate mean ± SD per timepoint and plate ID. When the radius was null at one of the timepoints involved in the interval, growth rate was not calculated. Therefore, only two growth rate values are shown for Plate 11 at the first interval. (**c**,**e**) Estimation of appearance time from radius at 24 h by linear extrapolation for the three colonies highlighted in (a) for each plate. The dashed line represents R_thresh_ (200 µm). The usage of either the same GR_max_ (65 µm/h) for all colonies, or individual colony GR_col_ leads to different results. (**d**,**f**) Bland-Altman^23^ comparison plots of the appearance time (t_app_) determined by full time-lapse analysis (real t_app_) and the appearance time estimated by linear extrapolation (predicted t_app_) with GR_max_ or GR_col_ respectively. The x-axis is the mean of the two measurements (obtained by full time-lapse analysis and linear extrapolation). The y-axis is the difference between the real t_app_ and the estimated t_app_. Positive values indicate underestimation and negative values an overestimation of appearance time by the linear extrapolation method. The dotted line is the average bias of the linear extrapolation method and the two dashed lines are showing the lower and upper limits of agreement.

To allow users to evaluate the validity of these two assumptions, or to estimate a reference growth rate if a time-lapse imaging setup is unavailable, ColTapp proposes a functionality to analyze a sequence of endpoint images (Supplementary text 7.1 and 7.2). By taking endpoint images at multiple timepoints and performing colony detection at each timepoint one can create a timeseries of colony radius. In Fig. 3a, we exemplify this functionality by analyzing independently four frames extracted from the time-lapse image sequences of three plates. Two of these plates belong to our demonstration dataset (plate 18 and plate 11, Supplementary Fig. S9, S10). With the third plate, we illustrate differences in growth rates, as it includes colonies from both a *S. aureus* laboratory strain and a *S.aureus* small colony variant clinical isolate, which exhibits a slow growth rate (plate MX, Supplementary Fig. S8). ColTapp computes the slope between colonies’ radii at different timepoints (Eq. 1), which results in an individual colony radial growth rate GR_col_ for each time interval (Fig. 3b).

This time-series analysis enables assessment of the entry time to saturation phase, identified by a decrease in growth rate (Fig. 3b). Moreover, should colonies have different growth rates, the individual colony radial growth rate can be used to estimate the appearance time, rather than a fixed reference growth rate for all colonies (Fig. 3c–f). Using individual colony growth rates increased overall agreement between appearance times determined by the full time-lapse analysis and appearance times estimated from endpoint images (Fig. 3d,f).

However, note that using individual colony growth rates for appearance time estimation is appropriate to correct for differences in size which are independent from saturation, for example when a subpopulation on the plate has mutations affecting growth rate or a different genetic background (e.g. Plate MX in Fig. 3). Should colonies be past the linear growth phase, growth rate estimations are inaccurate, thus appearance time cannot be accurately predicted (e. g. Plate 18 at 24 h in Fig. 3). In these cases, one needs to take saturation into account. We introduce an approach to density-based corrections of saturation in the next section.

### Response to density

**Figure 4:**
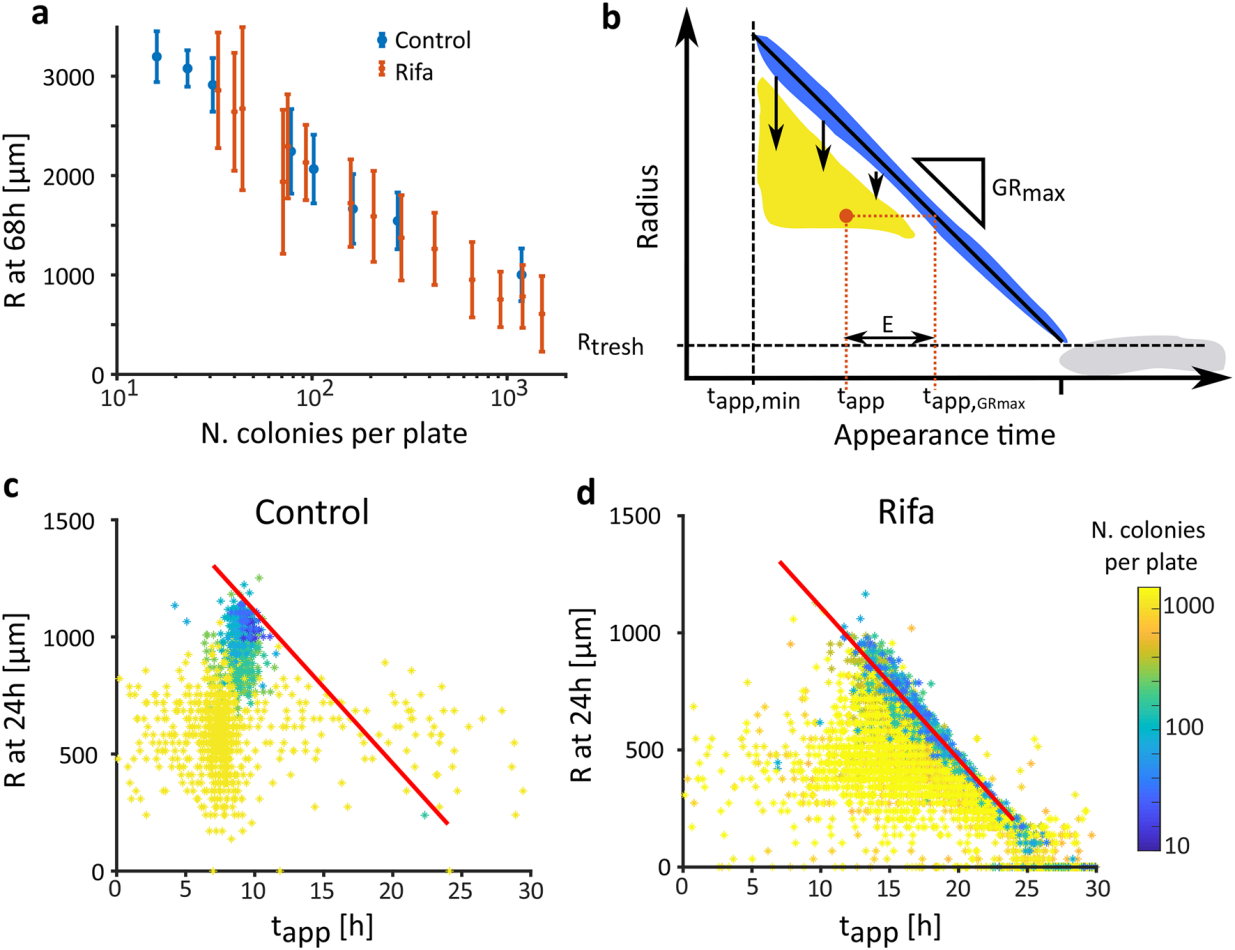
Plate density affects colony size. (**a**) At a given time (here 68 h), colonies on a dense plate have a smaller radius than colonies plated at low density. This effect is visible if colonies all grow at the same time (Control, blue) or after a delay (Rifa, orange). (**b**) This relationship between radius and appearance time at a given time can be visualized as a drop of the colony radius (yellow) from the ideal growth line (blue). An example of one colony is shown, represented by the orange dot. Its appearance time (t_app_) is given by its x-coordinate, and the appearance time estimated using a linear extrapolation with GR_max_ is given by the x coordinate of its projection on the ideal growth line (t_app,GRmax_). The error (E) is the difference between these two values along the x-axis. (**c**) In our control dataset, the linear relationship expected between the colonies’ appearance time and their radius (red line defined by GR_max_ = 65 µm/h) was lost when colonies grew on dense plates (here with warmer colors) and was heterogeneous within a plate at a given time. Here, at 24 h, while some colonies were still following a linear growth, other colonies from the same plate had already entered saturation phase. (**d**) The effect of plate density was also visible in the rifa dataset, with a larger variance in colonies’ appearance times and radii.

One typical problem for environmental or clinical samples is that the number of colony-forming-units per milliliter of sample is often unknown before plating, resulting in unpredictable colony densities on the plates. Yet, colony growth dynamics are affected by the presence of neighboring colonies, which compete locally for nutrients and are affected by the total number and proximity of colonies on the plate^16,24^. As soon as the effects of competition with neighboring colonies become strong enough^25^, a colony enters the saturation phase of its growth, meaning that its radial growth rate decreases over time until it reaches 0 (Fig. 2). Therefore, colonies on denser plates are expected to be smaller than colonies on less dense plates at a given point (Fig. 4a).

On top of this, if the plating is heterogeneous, not all colonies have access to the same amount of resources. They therefore differ in size within a plate as soon as they enter the saturation phase. Thus, experiments aiming to compare either colony size or appearance time are biased by colony density.

We explored the response to density using our purposely designed demonstration dataset of colonies formed by the *S. aureus* lab strain JE2 on Columbia sheep blood agar. Of the 22 time-lapse movies, eight were plated from a single liquid culture at exponential phase to obtain a homogeneously growing population (control dataset, Fig. 4a). The other fourteen were plated from another culture immediately after 24 h exposure to the antibiotic rifampicin (further referred to as rifa dataset, Fig. 4a). Rifampicin arrests bacterial cell growth by stopping protein synthesis, resulting in a bacterial population with a wide appearance time distribution^26^. In order to observe the impact of density on colony radius over time and elaborate a method to correct this bias, the replicates were plated at different densities over two orders of magnitude (ranging from 16 to 1509 colonies per plate) (Supplementary Fig. S9, S10).

The effect of high densities on the colony growth rate is shown on Fig. 4c,d displaying the colonies’ radii against their observed appearance times at a given timepoint. One may follow the evolution of this relationship over time (Supplementary movie 2). At low densities, colonies grow at the maximal linear radial growth rate, GR_max_, which depends on strain and culture conditions (here 65.0 µm/h, see section Appearance time and growth rate determination from time-lapse images). Colonies that deviate from the GR_max_ appear below the expected linear regression line (Fig. 4b). Therefore, when performing a linear extrapolation using GR_max_, the appearance time of colonies which are already in the saturation phase are overestimated (i.e. colonies on plate 18, Fig. 3d). The systematic error (E) introduced by saturation corresponds to the difference between t_app, GRmax_ (the result of the linear extrapolation with GR_max_) and the appearance time (t_app_) determined by the full time-lapse analysis (Fig. 4c):

2 $$t_{app} = t_{{app,GR_{max} }} - E.$$

The error depended both on the local density of the plates and on the age of the colonies on the plate. Colonies in denser plates (warmer colors) deviated more than colonies on less dense plates (colder colors). Also, at a given time point, colonies on plates with earlier appearance time (Fig. 4c) deviated more than the ones on plates with later appearance times (Fig. 4d).

### Density correction

Chacón et al.^16^ proposed that the amount of nutrients available to each colony on the agar plate can be approximated with Voronoi cell areas, obtained by drawing equidistant lines between neighbor colonies centers. The correlation between the radius at the time of observation and colony Voronoi cell area is time dependent and strongest at a late growth stage, as colonies approach their maximal size^16^.

**Figure 5:**
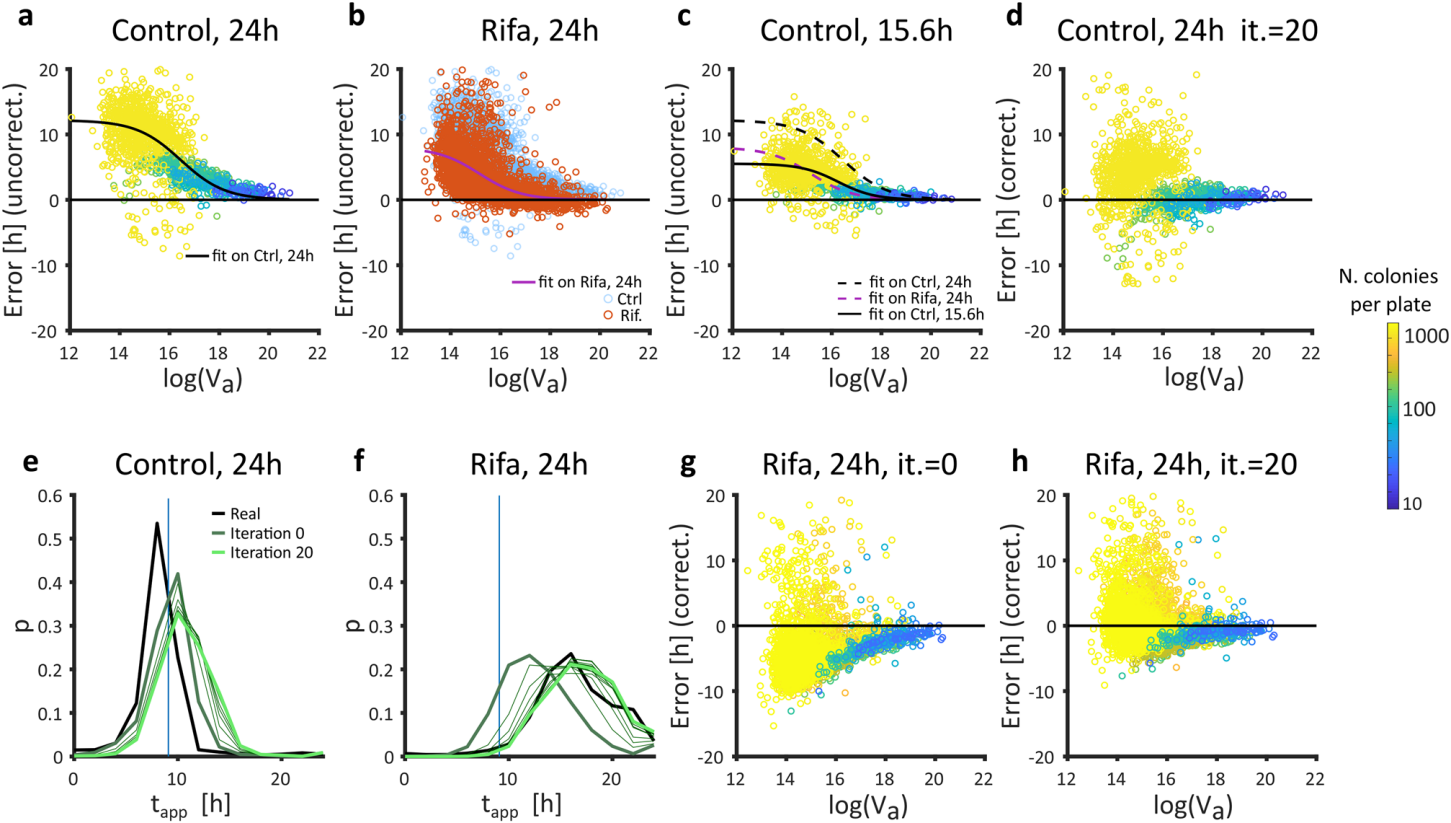
Iterative correction of density effects. (**a**) For a given timepoint (here 24 h, control dataset), the error between appearance time estimated by linear extrapolation and appearance time determined by the full time-lapse analysis correlates with the Voronoi cell area (V_a_) and can be fitted using a logistic model (black line). Each dot represents one colony, colored by density (log values on the color bar, left side of figure) same as in (**c**, **d**, **g**, **h**). (**b**) The relation E(V_a_) observed at 24 h on the control dataset (blue dots underlaid on this plot) depends on the median lag time. Here it can be seen that E(V_a_) observed at 24 h on the rifa dataset (red dots, fitted by the purple line) is systematically smaller. (**c**) Here the relation E(V_a_) observed at 15.6 h on the control dataset is shown. The fit of this data (black solid line) is very close to the fit of E(V_a_) observed at 24 h on the rifa dataset (dashed purple line). Dashed black line is the fit of E(V_a_) observed at 24 h on the control dataset. (**d**) The result of the iterative correction for the control dataset observed at 24 h (shown in (**a**) before iterative correction). (**e**,**f**). Visualization of the improvement of appearance time estimation by iterative corrections depending on time difference between median estimated t_app_ and the known median t_app_ of the control dataset (vertical blue line), yielding predictions closer to the appearance time determined by the full time-lapse analysis (real, bold black line). The initial guess (Iteration 0, bold dark green line) is corrected (thin lines) over 20 iterations to yield final corrected t_app_ (Iteration 20, bold light green line). Histograms of the probabilities p are binned in 2 h bins. (**g**,**h**) Estimated appearance time of the rifa dataset (shown without correction in **b**) is either corrected using E(V_a_) observed at 24 h on the control dataset (no iterations), or through 20 iterations (abbreviated it. on figure).

Aiming to use the Voronoi cell area (V_a_) to approximate the error in appearance time estimation introduced by saturation (E), we evaluated the mathematical relation between log(V_a_) and E in the control dataset. At 24 h, E was close to 0 for colonies with large V_a_ and increased for colonies with smaller V_a_ (Fig. 5a, we attribute negative values to noise in colony detection at very high density). The relation E(V_a_) was fitted with a modified logistic model (Fig. 5a):

3 $$E\left({V}_{a}\right)={E}_{max}-\frac{{E}_{max}}{1+{e}^{b\left(c-\mathrm{log}\left({V}_{a}\right)\right)}}$$

where E_max_, b and c are fitted parameters using the least square method. E_max_, depends on the control dataset. In theory, one could imagine that maximal error would increase indefinitely, but E_max_ exists because even for infinitely small values of V_a_, colonies still grow by using nutrients immediately below them, even though they do not receive flux from the sides. b and c are shape parameters for the transition from no error to E_max_.

As expected, the relation E(V_a_) was time dependent. When fitted through time, a linear increase of E_max_ was observed starting as soon as the first colonies enter saturation phase (Supplementary movie 3, Supplementary Fig. S11). In addition to being time-dependent, E(V_a_) was observed to depend on the general lag time of the plate: at a given timepoint, colonies originating from populations with a large median lag time (e.g. rifa dataset) were in an earlier phase of their growth as compared to colonies originating from populations without lag (e.g. control dataset) (Fig. 5b). Ideally, one would adapt the relation E(V_a_) to take into account the median lag time of the plate, so that the correction applied to the rifa dataset observed at a given timepoint (t_obs_) corresponds to E(V_a_) fitted on the control dataset at an earlier timepoint, when the colonies were still at that stage of growth.

The difference in appearance time between the control dataset (median appearance time: 9 h) and the rifa dataset (median appearance time: 17.4 h) was Δt_app_ = 8.4 h. The correction applied to the rifa dataset when observing colonies at t_obs_ = 24 h should thus correspond to E(V_a_) fitted on the control dataset at t_obs_-Δt_app_ = 15.6 h (Fig. 5c). However, the median appearance time is not known a priori when observing colonies on a plate at an endpoint.

Let us assume that we know the radius at 24 h but do not know the appearance time of the rifa dataset. We propose to use an iterative process to estimate the appearance time based on the knowledge acquired from the (strain and condition dependent) control dataset with known appearance time. An initial guess of the appearance time (t_app,i_) was obtained with Eq. (2) where E(V_a_) was fitted on the control dataset at t_obs_. Then the difference between the known median t_app_ of the control dataset and the median t_app,i_ of the observed rifa plate (Δt_app,i_) was calculated. A new t_app,i_ was estimated with Eq. (2) where E(V_a_) was fitted on the control dataset at t_obs_—Δt_app,i_, which in turn gave a new Δt_app,i_. This process was iterated until stabilization for a final estimate of t_app_ (Fig. 5d–h, Supplementary Fig. S11).

Note that the shown approach tended to overcorrect the appearance time of colonies on dense plates due to the nature of the data: fully saturated colonies have little information left regarding their appearance time since the radius does not change with time anymore. Thus, this correction cannot perform well if many colonies have reached late saturation phase (Supplementary Fig. S11). For this reason, in populations with very large variance of appearance time, one single image may not allow a proper appearance time estimation, especially if some colonies stop growing before the late ones appear. In such cases, we recommend using several pictures of the same plate at different timepoints in order to minimize possible biases in estimations of lag time distributions.

## Discussion

We developed ColTapp, a user-friendly application with a graphical interface. This application extracts biologically relevant data from images of microbial colonies including size, growth rate and appearance time as well as morphology descriptors and spatial metrics. Colony growth dynamics parameters are derived from time-lapse images. As it might not always be feasible to utilize high-throughput time-lapse imaging, ColTapp also includes a framework to estimate appearance time from endpoint images. Colony size at an endpoint is affected by colony density, resulting in a bias in appearance time estimation. Thus, we propose an iterative correction utilizing local plate density to moderate this bias. The correction is based on a control dataset and applied to a dataset with different growth characteristics (generalized long lag time).

In the past decades, the existence of phenotypic heterogeneity in bacterial populations has gained interest. Mathematical modeling of bacterial population dynamics in their ecosystems used to rely on average behavior. Advances in single cell microscopy and the development of models that can take into account the phenotypic state of thousands of individual bacteria shed light on the ecological function of phenotypic heterogeneity^2,27^. In medicine, while most bacterial infections can successfully be treated with antibiotics, treatment failure occurs even if the bacteria are not resistant to the antibiotics that are given. The existence of subpopulations unresponsive to treatment is thought to ultimately be the cause for these treatment failures.

Under standard laboratory conditions, the established methods to investigate phenotypic heterogeneity in bacterial populations are single cell microscopy and flow cytometry, but these methods are often expensive and technically difficult to setup and maintain. Moreover, many medical and environmental samples are too complex to study with these methods, either because they consist of complex substrates (e.g. viscous fluids) or they contain contaminating particles that may hinder proper quantification. The high fluctuation in quality and bacterial load in natural samples might also not justify the time and money investment required to carry those experiments out. In the case of microscopy, rare phenotypes are difficult to quantify if the setup does not allow the observation of very large number of bacteria. Therefore, plating bacteria on nutrient media offers a cheap and easy way to assess lag time heterogeneity, as previously proposed^4,5^. While time to growth resumption on undefined nutrient media reflects the dormancy state of the bacterial cells upon plating, one may be interested in following time to growth resumption of an active population upon plating on selective media. Indeed, this can be an opportunity to investigate the ability of a population to switch metabolic states (e.g. to a new carbon source)^28^.

Note that although colony formation can be seen as a good alternative to single cell behavior assessment, it is not a direct single cell observation. For example, clumped bacteria or an inhomogeneous spreading of cells on a surface, resulting in deposition of aggregates of cells instead of single cells on the agar, will likely result in an underestimation of single cell lag time. This needs to be kept in mind even when calibrating data to optimize analysis. Assessing the effect of the studied condition on clumping of bacteria might help avoiding false discoveries.

With ColTapp, we propose an easy to use graphical user interface, so that any laboratory, regardless of the technical equipment and image analysis expertise accessible, can approach single cell growth phenotypes by monitoring colony growth dynamics. Usage of colony size determined by image analysis to assess stressor effects is a long-established method^29^ and various end-user applications to facilitate colony counting and size assessment were developed over the years (e.g. a standalone GUI by Clarke et al.^30^ or an ImageJ plugin by Cai et al.^31^), of which OpenCFU is one of the best known applications^32^. Image analysis algorithms implemented by the various applications are usually very similar and consist of a combination of pre-processing steps to clean raw images, a variety of thresholding algorithms to derive binary images and optional watershed segmentation to finally count and measure isolated objects. Variations exist, with for example multiple rounds of pre-processing^33^ or a two-step colony detection algorithm for plate border and center^34^. Modern colony counter image analysis applications are using smartphones to acquire and process images all in one simple app^35,36^ or are starting to use the power of deep learning algorithms^37^.

OpenCFU was the best operating tool at the time of writing this manuscript, while many other programs are not maintained. We compared its performance to detect colonies with ColTapp, using our image dataset of colonies from multiple species plated on multiple media (Table 1, Supplementary Fig. 5, Supplementary Table S2). The average ratio between found colonies and actual colonies on an image (not accounting for false positive or false negative detections) was 0.75 (SD = 0.26) indicating a general underestimation of colony numbers. ColTapp on the other hand has ratio of 1.09 (SD = 0.28) between initially detected and actual colonies. ColTapp, on average, overestimates number of colonies. OpenCFU has no option for manual correction of detected colonies which limits the usability of it.

**Table 1:** Comparison with other colony image analysis tools.

	Availability		Data extracted from colonies on images					Aim
	Code	GUI	Count	Position	Size	Growth kinetics	Additional characteristics	
Levin-Reisman et al. 2010 (ScanLag)	Yes	No	Yes	Yes	Yes	Yes	None	Analysis of colony time-lapse images
Geissmann 2013 (OpenCFU)	Yes	Yes	Yes	Yes	Yes	No	Morphology descriptors, number of adjacent colonies	Analysis of colony endpoint images
ColTapp	Yes	Yes	Yes	Yes	Yes	Yes	Morphology descriptors, spatial metrics	Analysis of colony endpoint and time-lapse images

Here, we qualitatively compare ColTapp with two tools dedicated to image analysis of pictures of bacterial colonies grown on solid agar. We summarize the availability of code and a graphical user interface (GUI), the output data provided (colony counts, position, size and growth kinetics, i.e. appearance time and linear radial growth rate) as well as the aim of the three tools. For details and more publications, see main text.

The above mentioned applications (Table 1) are meant to analyze endpoint images, which do not capture colony growth dynamics. Levin-Reisman, et al.^5^ released the ScanLag code package to analyze colony time-lapse images. Yet, although multiple groups use this type of analysis for their research^7,15,16,38^, there is no comprehensive and flexible application including a graphical user interface currently available for this purpose. ScanLag works best on a specific imaging setup and we were not able to compare it with ColTapp. Therefore, by bringing together the standard endpoint analysis with time-lapse image analysis, our user-friendly ColTapp fills a gap in the collection of existing dedicated image analysis tools.

As for any image analysis tool, the quality of the analysis will highly depend on the quality of the images themselves. We recommend users to maintain homogenous lighting, avoid light flares and reflections and create good contrast using background modifications and proper focusing. ColTapp is flexible enough to allow processing of colored and grayscale images acquired either through dedicated platforms or with simple camera solutions. For endpoint images, this can be best achieved using a dedicated white box with diffused, indirect light, but recent phone cameras can also give decent results on a bench.

We propose an analysis of the linear radial growth phase, assuming a colony has already reached this stage upon appearance. It is not difficult to observe the initial exponential part of the colony growth (occurring before a colony reaches a radius of ~ 100 µm) by using commercially available photographic macro lenses (Supplementary Fig. S7, see methods). However, one should be aware that defining appearance time with a linear extrapolation is incorrect in this case as the exponential growth phase is actually observed and should be taken into account. Similarly, our estimation of appearance time from endpoint images assumes that colonies are in the linear growth phase.

As soon as the steady flow of nutrients sustaining colony growth decreases (timing is dependent on proximity of competing neighbors and the total number of colonies on the plate), the colony enters saturation phase, which impacts the linear appearance time estimation. Density may even affect the observed appearance time if colonies enter saturation phase before they are visible^5^. However, in the presented *S. aureus* dataset (including extremely dense plates), only a marginal correlation was observed between colony appearance time and density (Supplementary Fig. S12). Colony growth is a complex biological process and different species on different growth substrates will respond differently to density^16^. For this reason, it is impossible to propose a universally valid method to take density into account. Nevertheless, the explored correction based on spatial metrics, which can easily be exported with ColTapp, might be used as a starting point to develop suitable correction methods for the investigated species.

We wrote the ColTapp application using the classical programming language MATLAB, and the code is designed as a modular shell that can host further image analysis methods that may meet specific needs, while benefiting from the easy to operate graphical interface. Typically, we envision that the introduction of more sophisticated morphology descriptors could facilitate the study of intra- and inter-species colony interactions.

## Materials and methods

### Hardware and software details

We programmed the ColTapp application with MATLAB 2020a (MathWorks). Very preliminary code of the GUI and time-lapse radius tracking was proposed earlier^8^. All algorithm accuracy and computational efficiency tests were performed on a computer with Windows 10 OS, i7-8700K @4.70 GHz, 32 GB RAM.

We used external code acquired from the File Exchange server of MathWorks for certain functions. Namely, these were a Voronoi tessellation function by Sievers^39^, an image zoom and pan function by Cabrera^40^, multiple directory acquisition function by Cannell^41^, a smoothing function tolerant to missing values by Pittam^42^ and a function to fit a circle through three points by Malyuta^43^.

Additionally, we used ggplot2^44^ within R and R Studio^45^ as well as Inkscape (www.inkscape.org) for certain figures.

### Image acquisition

ColTapp does not rely on a specific image format or quality. Achieving homogenous lighting is undoubtedly critical for successful image analysis, but image acquisition itself can be operated using any kind of device. For correct image capture (Supplementary text 2.1), the camera should be positioned perpendicular to the plate. For time-lapse imaging, acquisition triggering at defined time intervals is necessary. Typical systems include scanners, dedicated applications and cameras or commercially available consumer-grade camera, provided they include a time-lapse mode or can be triggered by an external controller.

The time-lapse series presented in this paper were generated acquiring images every 10 min for ~ 70 h, resulting in series of either 410 or 423 images. Monitoring colony growth for this duration ensured that bacteria experiencing long lag time became eventually visible and that the colonies reached the saturation phase on all plates to permit our analysis of density.

Plates were either captured individually with Canon EOS 1200D reflex cameras generating .jpg files of size 5184 × 3456 pixels triggered through an Arduino controller (Fig. 1a) or four plates at a time with Basler acA5472-17um 20MP cameras (Fujinon Objectives CF16ZA-1S 16 mm/1.8M37.5 × 0.5) generating .tiff files of size 5472 × 3648 pixels triggered with Basler’s pylon software suite (Version: 6.0.1). The spatial calibration factor was around 1 pixel to 25 µm using the Canon EOS 1200D and 1 pixel to 50 µm using Basler acA5472-17um 20MP cameras. When using an EF100mm f/2.8L IS USM macro lens on the Canon camera, we obtained a 1 pixel to 5 µm spatial calibration factor. The plates were laid on a black background surface upside-up below the cameras and the whole system was set inside an incubator at 37 °C. To prevent drying out of agar plates and airborne contamination, lids were kept on the plates. To avoid condensation, lids were pre-warmed to 37 °C. Best image analysis quality was observed when special attention was brought to the illumination method: indirect, homogeneous white illumination was obtained by covering the incubator walls with white paper and using a LED based light source (Philips Hue Lightstrips Basis, operating in warm-white mode) emitting minimal heat (temperature of LED aluminum encasing: 45.1 °C ± 2.4 °C (mean ± standard deviation, n = 7); temperature of agar plate surface: 37.5 °C ± 0.51 °C. Temperature measured with a Stanley STHT0-77365 infrared thermometer). Endpoint images were acquired manually with a Canon EOS 1200D reflex camera and lids were temporarily removed. Plates were placed in a custom-built box with light diffusing sides and a fixed plate- and camera-holder.

### Colony detection algorithm implementation

**Figure 6:**
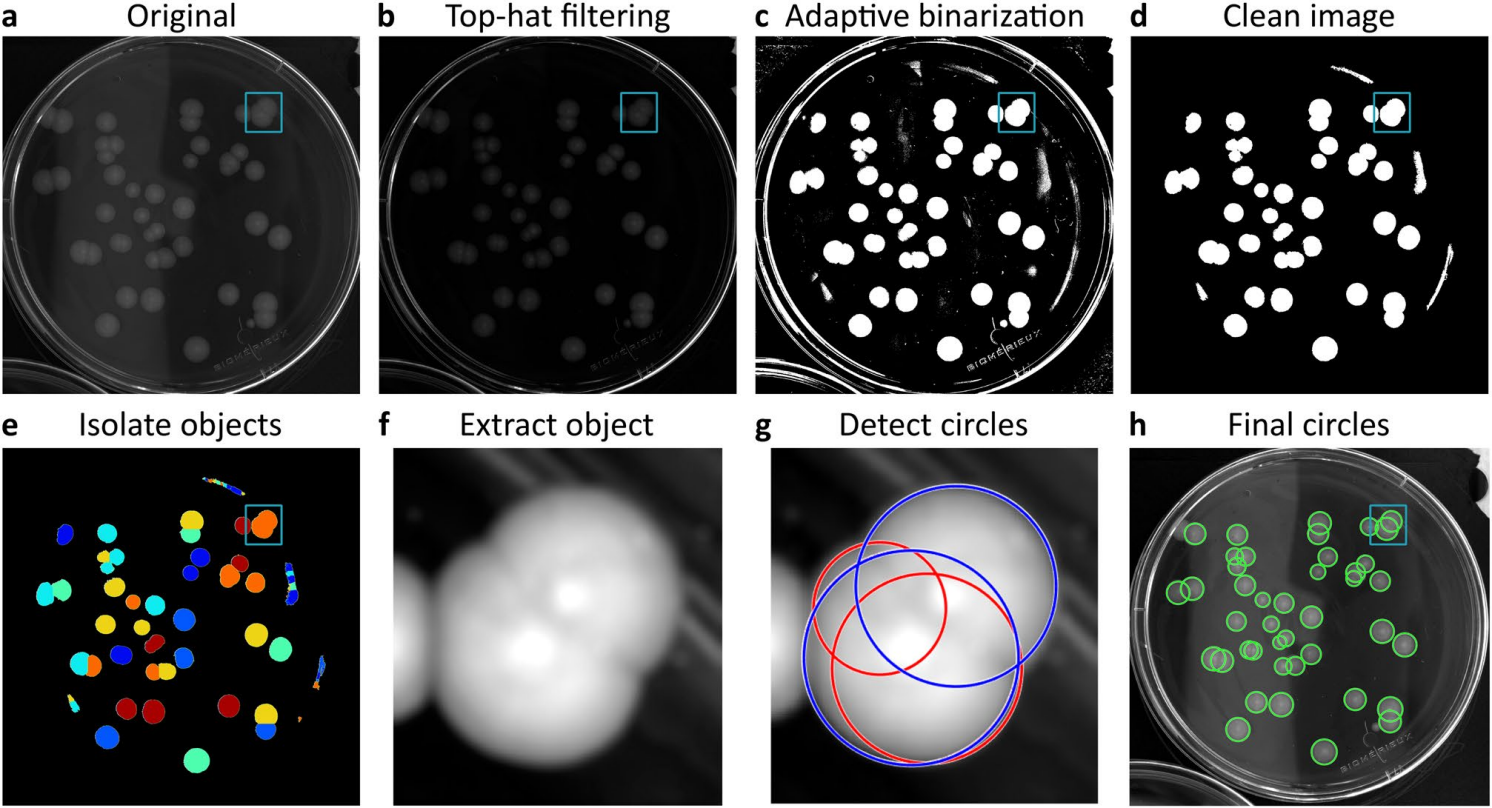
Image analysis steps for colony detection. (**a**) Original grayscale image. (**b**) Top-hat filtering of image in (**a**). (**c**) A binary image is obtained by adaptive local thresholding. (**d**) Noise is filtered out of binary image. (**e**) Distance transformation and watershed segmentation are used to identify isolated objects. (**f**) Sequentially, each minimal sized image containing an isolated object is extracted. (**g**) Potential circles are detected with a two-step Hough transform (all circles). A series of automatic quality control functions are used to discard false positive circles (red circles). Only circles passing the quality control are kept (blue circles). (**h**) Final detected circles. The small rectangle in (**a**,**b**,**c**,**d**,**e**,**h**) marks the region of the extracted object shown in (**f**, **g**).

Colony detection uses a series of image analysis operations starting from a grayscale image (Fig. 6a) and depends on user-specified minimal and maximal expected radius of colonies (see Supplementary text 3.1 and 3.2 for additional pre-processing possibilities). ColTapp uses top-hat filtering with a disk-shaped structuring element based on the minimal expected radius to reduce lighting gradients and other inhomogeneities of the agar background (Fig. 6b). Local adaptive thresholding is then used to create a binary image (Fig. 6c). This image is cleaned from artifacts and unwanted objects by discarding small and big isolated objects (threshold defined by minimal and maximal expected radius, respectively). Objects with a low extent (foreground/background pixel ratio), are discarded as well (Fig. 6d). Next, distance transformation is used to derive local minima, which are subsequently filtered by minima imposition. Overlapping colonies are separated with watershed segmentation (Fig. 6e). Sequentially, ColTapp extracts images containing an isolated object (img_crop_) from the top-hat filtered grayscale image and further performs circular filtering and contrast enhancement (Fig. 6f). Objects are discarded if the proportion of low intensity pixels within img_crop_ is above a threshold defined by the median intensity of all objects. A two-step circular Hough transform is applied to img_crop_ by the *imfindcircles* function^46,47^ available in MATLAB to detect all probable colony circles within the given radius range (all circles in Fig. 6g). False positive circles may be detected (red circles in Fig. 6g) and sequential quality control functions are used to exclude these. Circles are excluded based on their distance from the image boundaries of img_crop_, proportion of pixels categorized as foreground within the circle, proportion of overlap with each other, distance between each other and relative fitting quality. After every img_crop_ is processed, a final quality control round is deployed to remove circles with extremely close centers (Fig. 6h). Several parameters involved in circle detection and quality control can be tuned to potentially improve performance (Supplementary text 3.2). In addition, the user may intervene directly to correct the results (Supplementary text 3.3).

In *Endpoint* mode, colony detection is done independently on each frame. In *Timelapse* mode, it is done on a single frame, as the colony radius can subsequently be tracked over time.

We tested the accuracy and computation speed of the colony detection algorithm with 30 images acquired with different cameras, including smartphones (Supplementary Fig. S5). Images of the bacterial species *S. aureus*, *Escherichia coli*, *Staphylococcus epidermidis*, *Pseudomonas putida*^48^*, Acinetobacter johnsonii*^48^ and *Alteromonas macleodii*^49^ as well as bacteriophage plaques on *E. coli*^48,50^ using the standard agar media necessary for optimal growth of the respective species (Colombia sheep blood, tryptic soy broth, lysogeny broth, marine broth, etc.) were included. ColTapp successfully detects colonies from various bacterial species on distinctly colored agar plates. Adapting imaging conditions towards homogeneous light and reduced reflections can reduce the amount of manual correction required (Supplementary text 3.4, Supplementary table S2 and Supplementary Fig. S5).

### Radius tracking algorithm implementation

**Figure 7:**
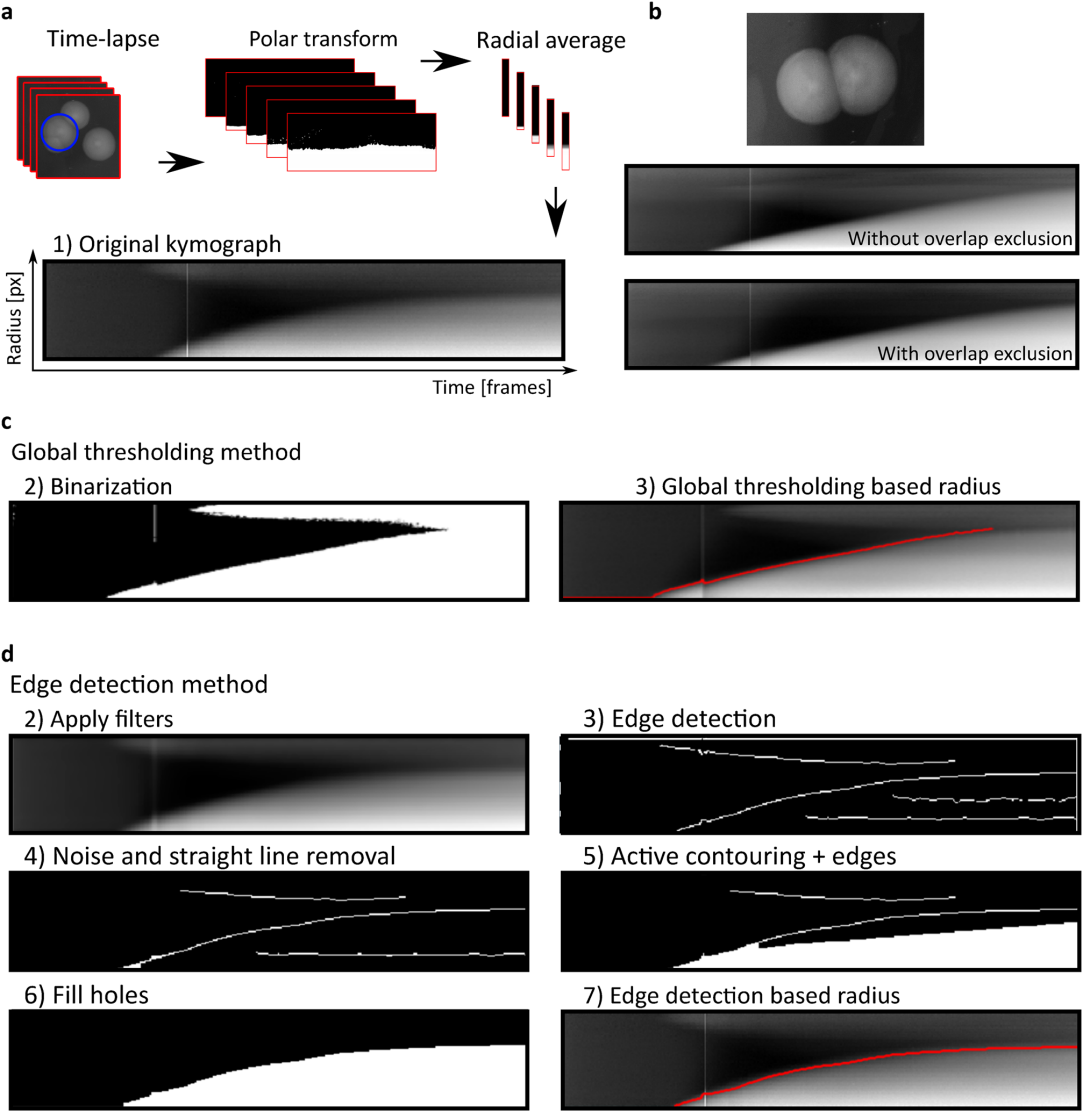
Time-lapse processing. (**a**) A polar transformation is applied to grayscale sub-images of each colony and intensity values are averaged over the radius for each frame in the time-lapse. These radial average intensity vectors are combined into a kymograph representing colony radius growth. (**b**) Overlapping colonies result in blurry regions in the top part of the kymograph. The angle range corresponding to overlapping colonies is excluded to reduce this effect. Kymographs can be processed either by (**c**) *Global thresholding* or (**d**) *Edge detection* method. (**c**) In the *Global thresholding* method, a threshold is applied to a filtered version of the kymograph to derive a binary image. (**d**) The *Edge detection* method uses a series of filtering steps, edge detection algorithms and morphological operations to optimize the binary image.

Executing the above described colony detection method on each frame to generate time-lapse data of growing colonies creates several potential problems: (1) manual correction would be tedious, (2) colony to colony center matching between frames needs high accuracy, (3) radius range search of ColTapp is not time dependent, (4) the colony detection algorithm does not operate well with an expected minimal radius below 3 pixels. This would impose a detection limit. Therefore, ColTapp proposes to initially detect all colonies on a frame corresponding to a late timepoint, which is set as the *reference frame*. Once colonies have been detected on a late frame of the time-lapse series, their radius is tracked on the previous frames. For each time frame, ColTapp extracts sub-images of each colony from the main image. Intensity of each sub-image is scaled based on minimal and maximal intensity values of the last image of the time-lapse. These sub-images are then unwrapped with a polar transformation (Fig. 7a). The placement of the colony center position is important in this step since a misplaced center’s distance to the edge of the colony is not equal to the radius. Image drift correction as well as center correction functions are implemented in ColTapp to improve centering (Supplementary text 5.1 and 5.2). Each transformed sub-image is averaged into a single intensity vector representing the intensity gradient from the colony center towards its edge. This averaging step reduces the noise introduced by slight off-centering of colonies and reduces file sizes. Finally, all vectors resulting from the corresponding sub-images of each time frame are combined into a kymograph, which represents the colony radial growth as a curve in intensity (Fig. 7a). The kymograph’s highest contrast edge corresponds to the colony radius over time. Since the presence of neighboring colonies would create blurry regions in the top of kymographs and thus hinder edge detection (Fig. 7b), ColTapp ignores the angle ranges corresponding to adjacent colonies for kymograph creation (Supplementary text 5.3).

In order to detect the kymograph edge to derive colony radius over time, two methods are implemented: *Global thresholding* and *Edge detection*. Both methods first apply an automatic contrast enhancement function, then a pixel-wise adaptive low-pass Wiener filter^51^ followed by a circular averaging filter (pillbox) to the kymograph to derive a smoother image. Additionally, a low-pass threshold for maximal intensity may be applied.

These pre-processed images are then further transformed with either a *Global thresholding* or *Edge detection* method to create binary images. The default *Global thresholding* method uses Otsu’s method^52^ to derive a global threshold from which a user-defined constant is subtracted to prevent biasing towards background (Fig. 7c).The *Edge detection* method uses a series of edge detection and morphological operations (Fig. 7d). In brief, the method by Canny^53^ is used for initial edge detection. Subsequently, small isolated foreground pixels are removed with area opening operations. Straight vertical and horizontal lines, typically originating from light artifacts and particles on the agar respectively, are removed. Two iterative morphological closing operations are used with line- and disk-shaped kernels, respectively. Additionally, active contouring using the Chan-Vese method^54^ is applied to the pre-processed kymograph to derive a second binary image with a filled region of probable colony area. The two binary images are merged, and morphological closing is performed to close potential gaps to successfully fill connected regions afterwards. A final morphological opening is applied to remove jagged edges. The fully connected object located in the lower right side of the kymograph (corresponds to end of time-lapse and close to colony center) is kept as the only foreground object in the binary image.

ColTapp includes automatic error detection and manual correction tools for the kymograph derived radius growth curves (Supplementary text 5.4, Supplementary Fig. S13).

The *Edge detection* method is more complex and is suggested when the default *Global thresholding* method fails. Switching to the *Edge detection* method for the incorrect growth curves resulted in a reduction of the number of growth curves requiring further manual correction to 3.1% (SD = 2.7%) (Supplementary text 5.5, Supplementary Table S3). Generally, computational time increases with the number of frames in a time-lapse series, number of colonies to track, image file size and final colony size (Supplementary text 5.5, Supplementary Table S3).

### Bacterial strains and cultivation

To generate our 22 demonstration time-lapse movies (Supplementary Fig. S9, S10), the *Staphylococcus aureus* strain JE2 was grown overnight at 37 °C in shaking (220 rpm) test tubes containing tryptic soy broth (TSB, Difco), inoculated from a single colony picked from blood agar plates. These cultures were diluted to an optical density (600 nm) of 0.2 in fresh TSB containing rifampicin (1 µg/ml) to generate the Rifa dataset and without rifampicin to generate the control dataset. After 24 h rifampicin exposure, cultures were pelleted (10,000 g, 3 min), resuspended twice in phosphate buffered saline solution and then plated on blood agar plates (Columbia, 5% sheep blood, BioMérieux) at dilutions ranging from undiluted to a 10^–4^ dilution in 2/3rd dilution steps. For the control dataset, after 2 h of incubation in TSB (corresponding approximately to mid-exponential growth phase), bacteria were serially diluted (2/3rd dilution steps) and plated on blood agar plates.

To generate the extra time-lapse movie of a plate including colonies from two different genetic backgrounds, the *S. aureus* strain Cowan I and a *S. aureus* small colony variant clinical isolate were picked from a plate, set to an optical density (600 nm) of 0.5, diluted to 10^–6^. 50 µl of each of these dilutions were plated on the same blood agar plate.

To generate the 30 images on which we assess the performance of the colony detection algorithm (Supplementary Fig. S5) the strains and growth conditions specified in Supplementary table S1 were used.

## Supplementary information

Supplementary information

## Data Availability

The ColTapp MATLAB source code and standalone executable (macOS/Windows) can be found at https://github.com/ColTapp under the GNU General Public License 3 and freely downloaded, together with a package of demo images, the code to trigger cameras with an Arduino board and experimental data analysis files. Raw images are accessible on Figshare at 10.6084/m9.figshare.12951152.v1.
