# Author Correction: Efficient microbial colony growth dynamics quantification with ColTapp, an automated image analysis application

**DOI:** 10.1038/s41598-021-85033-8

**Published:** 2021-03-09

**Authors:** Julian Bär, Mathilde Boumasmoud, Roger D. Kouyos, Annelies S. Zinkernagel, Clément Vulin

**Affiliations:** 1 Department of Infectious Diseases and Hospital Epidemiology, University Hospital Zurich, University of Zurich, Zurich, Switzerland; 2 Institute of Medical Virology, University of Zurich, Zurich, Switzerland; 3 Institute of Biogeochemistry and Pollutant Dynamics, ETH Zurich, 8092 Zurich, Switzerland; 4 Department of Environmental Microbiology, 8600 Eawag, Dubendorf, Switzerland

Correction to: *Scientific Reports* 10.1038/s41598-020-72979-4, published online 30 September 2020

This Article contains errors, where three Supplementary Information movies are omitted from the original version of this Article. The correct Supplementary Information movie files are linked to this correction notice.

Consequently, in the Results section under the subheading ‘A user‑friendly application implementing image‑ and downstream analysis’,

“In the following sections, the downstream analyses to derive growth parameters from the resulting data are demonstrated on a dataset of 22 time-lapse movies of *S. aureus* colonies growing on blood agar plates, designed to include both homogeneous and heterogenous colony lag time and various colony densities.”

should read:

“In the following sections, the downstream analyses to derive growth parameters from the resulting data are demonstrated on a dataset of 22 time-lapse movies of *S. aureus* colonies growing on blood agar plates, designed to include both homogeneous and heterogenous colony lag time and various colony densities (Supplementary movie 1).”

## Supplementary information

Supplementary movie 1.

Supplementary movie 2.

Supplementary movie 3.

