# Supplementary material for: Efficient microbial colony growth dynamics quantification with ColTapp, an automated image analysis application
Source: Sci Rep. 2020 Sep 30;10:16084. doi: 10.1038/s41598-020-72979-4 (PMC7528005; doi:10.1038/s41598-020-72979-4)
Supplement: Supplementary file 1 — Supplementary information [file 41598_2020_72979_MOESM1_ESM.pdf]

Supplementary Information for

Efficient microbial colony growth dynamics quantification with ColTapp, an automated image analysis application

Julian Bär[a†], Mathilde Boumasmoud[a†], Roger Kouyos[a,b], Annelies S. Zinkernagel[a], Clément Vulin[a,c,d*]

[a] Department of Infectious Diseases and Hospital Epidemiology, University Hospital Zurich, University of Zurich, Zurich, Switzerland

[b] Institute of Medical Virology, University of Zurich, Switzerland

[c] formerly: Institute of Biogeochemistry and Pollutant Dynamics, ETH Zurich, Zurich 8092, Switzerland.

[d] formerly: Department of Environmental Microbiology, Eawag, Dubendorf 8600, Switzerland.

[†] These authors contributed equally to this work

[*] Corresponding author: clement.vulin@usz.ch

Table of Contents

Extended description of the implementation *3*

1 Graphical user interface **3**

2 Analysis setup **3**

2.1 Spatial calibration 3

2.2 Area of interest 3

3 Colony detection **4**

3.1 Color image conversion 4

3.2 Algorithm parameters 4

3.3 Manual corrections 4

3.4 Algorithm performance 5

4 Colony characteristics **6**

4.1 Morphology descriptors 6

4.2 Spatial metrics 6

5 Tracking radius over time **7**

5.1 Drift correction 7

5.2 Colony centers correction 7

5.3 Overlapping colonies 8

5.4 Radial growth curve correction 8

5.5 Algorithm performance 8

6 Reference growth parameters definition **9**

7 Sequence of endpoint images **9**

7.1 Delayed colony detection 9

7.2 Sequence of endpoint images 9

8 Options **10**

9 Data export **10**

Figures *12*

Tables *25*

Movies *29*

Extended description of the implementation

1 Graphical user interface

Global operating buttons to load images and save data are in the top left part of the graphical interface (Fig. S1-a) and information about currently operating functions and text feedback in the top right part (Fig. S1-b). Upon loading a folder containing images to analyze, the user needs to define the operating mode: *Time-Lapse* (TL) or *Endpoint* (EP) mode, which cannot be changed for further analysis. Images in a folder are sorted with a natural sorting algorithm by name and assigned a frame number (from 1 to number of images in the folder). The currently active frame is visible at the center of the graphical user interface (Fig. S1-c). The user can switch between frames by moving the slider, typing the number of the desired frame (Fig. S1-d), navigating with the buttons on the sides of the image or pressing left/right arrow keys. The user can choose to display available data on the current frame in the *Options* menu, which also contains additional parameter configuration and gives access to other functorialities (Fig. S1-e). The main functionalities of ColTapp are grouped in three tabs on the left of the interface: *Detect*, *Main* and *Visualize* (Fig. S1, f g and h respectively). Two versions of the main panel are shown (Fig. S1-g) because the panel is dynamically adapted depending on the mode chosen by the user (TL mode, EP mode). Data previewing is possible through functions accessible within the *Visualize* tab (Fig. S1-h) with slightly differing options for TL and EP mode. Size visualizations can either be done in pixel or micrometer units (*Options*). Radius distributions can be displayed for a user-defined frame of time-lapse or a user-defined image of a set of EP images while also giving the option to combine all images of a given folder into one distribution. In TL mode, the radial growth curves can be visualized. Time can be set as either frame or micrometer (*Options*). Appearance and growth rate distribution visualizations are also implemented. The number of bins used for distributions can be set within *Options*.

2 Analysis setup

2.1 Spatial calibration

The program proposes to define a pixel to µm spatial calibration factor. The user may either directly enter the factor if known, or define the diameter of a circle, typically the agar plate (automatically detected), or any reference line of known length on the image.

2.2 Area of interest

Automatic colony detection can be narrowed to the defined boundaries of an area of interest (AOI). Users can select the plate as AOI or draw a custom polygon on the image. Using an AOI is

suggested to reduce computational time and reduce false positive detection outside the boundaries of e.g. an agar plate. In time lapse mode, both the spatial calibration factor and AOI are propagated from the active frame to the other frames. In EP mode, these variables are only applied to the current image. This can then be manually propagated if all images in a given folder were acquired with the same setup in *Options*: *Apply calibration factor/AOI to all frames*.

3 Colony detection

3.1 Color image conversion

ColTapp offers 16 methods to transform a color image into grayscale. Using one of the three RGB channels is computationally quick and generally precise enough, but in some special cases the user may want to use a different conversion as for example a transformation to other classical color spaces (e.g. CIELAB) and using one of the generated channels. The inbuilt MATLAB function rgb2gray which uses a weighted image conversion, retaining information of all three color channels, is also available.

3.2 Algorithm parameters

A selection of parameters involved in circle detection and quality control can be tuned to potentially improve performance, although we tested the default parameters values on a selection of images and reported high accuracy. The range of expected radius is an exception: it greatly affects computational speed and accuracy of the algorithm. We thus suggest to always define it, setting it as narrow as possible. The range boundaries (expected minimal and maximal circle radius in pixels) can be either derived automatically from the smallest and biggest circle drawn on the currently displayed image with the function *Define radius range* or directly specified within the *Options* dialog. The following other parameters can be modified: the relative size of img_{crop}, minimal distance from img_{crop} boundaries, a bias towards foreground classification as well as the minimal proportion classified as foreground within a circle. Maximal proportion of a circle overlapping with one other circle as well as minimal distance and radius difference are modifiable. Additionally, the user can set maximal total area of a circle overlapping with any other circle and define the final minimal distance between any circle.

3.3 Manual corrections

Addition of non-detected colonies and false positive removal is possible through simple mouse-guided operations. Additionally, polygons can be drawn on the image to clear out entire zones from found circles. In TL mode, it is important for downstream analysis to check if none of the detected circles is composed from two (or more) colonies which merged during the time-lapse

imaging. These should be removed and replaced with the appropriate number of circles with corresponding centers. This control step is needed to be done manually.

3.4 Algorithm performance

For the example shown in Fig. 6, a 1956x1936 pixels sized grayscale image displaying 42 *Staphylococcus aureus* colonies after 70.5 h of growth on Columbia sheep blood with an average radius of 53 pixels, the computation from raw grayscale image to isolated objects took 1.57 s and the circle detection and quality controls took 0.98 s. Note that computational speed is inversely proportional with image size, average colony size and number of colonies. A high proportion of plate area covered with colonies with high amount of overlap will generally yield poorer detection results and require manual correction (Supplementary table S2).

Computation efficiency and accuracy of the colony detection algorithm was tested with a selection of 30 differing images acquired with different cameras, including smartphones, (Supplementary Fig. 4). Images of the bacterial species *S. aureus*, *Escherichia coli*, *Staphylococcus epidermidis*, *Pseudomonas putida, Acinetobacter johnsonii* and *Alteromonas macleodii* as well as bacteriophage plaques on *E. coli* [1,2] using the standard agar media necessary for optimal growth of the respective species (Colombia sheep blood, tryptic soy broth, lysogeny broth, marine broth, etc.) were included (See Supplementary table S1 for experimental details). On this collection of images, which displayed colonies with an average radius of 27 pixels (SD = 14 pixels), colony detection took on average 0.053 s (SD = 0.035 s) per detected circle. Detailed values for each image are shown in Table S2. Generally, smaller image size and a narrower range of expected radii improve computational speed but may negatively impact accuracy.

Generally, ColTapp accurately detected colonies when the area of interest, expected radius range and the method for conversion of a color image into a grayscale image were set for each image separately (SI text 2.1). Note, that although colonies are usually lighter than the background, phage plaques are darker than the bacterial lawn: an option to find darker-than-background circles is available.

Overall, false positive rate varied substantially, ranging from 0% to 55.6% (median = 3.54%) (Supplementary Fig. S5A). Almost all images with false positive rates exceeding 5% had most of their wrongly detected circles in clusters (marked with blue symbols in Supplementary Fig. S5A), usually in areas with lighting artifacts (for example the triangular-shaped light reflection on image 30). These clusters can be cleared with a few clicks and do not pose a problem in our opinion. The one other case with a high false positive rate was an image displaying colonies of the marine bacterial species *Alteromonas macleodii* and a lot of chitinous debris, forming particles which were only marginally different in size as compared to the bacterial colonies. Therefore, false

positive rate should not exceed 5% if ideal imaging conditions are maintained. Additionally, we did not observe any correlation between false positive rate and total number of colonies on a plate (Supplementary Fig. S5A).

The false negative rate varied between 0% and 21.7% (median = 4.06%) and slightly increased with total number of colonies on the plate (Supplementary Fig. S5B). Some of the images with high false negative rates had strong lighting gradients, high size heterogeneity or a high number of overlapping colonies all of which can decrease performance of ColTapp's colony detection algorithm.

4 Colony characteristics

4.1 Morphology descriptors

ColTapp can extract basic colony morphology descriptors (including color, outline and texture) from each frame for downstream analyses, such as species identification or observation of mutation-induced phenotypes linked with altered growth on agar plates. Color values can be extracted from the entire colony or from the center (within a 5 pixel radius) and texture is calculated either as standard deviation of the color or image entropy[3] (Supplementary Figure 4). The length of the perimeter of colonies as well as the standard deviation on the average radius are metrics useful to categorize colonies based on the shape of the border. We also propose to export the mean color of the halo around colonies as this could be useful for colorimetric assays[4] or quantification of hemolysis capacities of the colony.

4.2 Spatial metrics

For post-processing steps such as density correction or the study of colonies interactions, the user can export typical spatial metrics calculations. Interactions between neighboring colonies are mostly occurring through the diffusion of small molecules through the agar plate matrix. The diffusion in such 3D gels can sometimes be approximated to be two-dimensional, and basic solutions of the diffusion equations will give interactions based on the distance D between colonies either as $\frac{1}{D}$ or $\frac{1}{D^2}$ depending on how the diffusion equation is solved. In their study to estimate parameters influencing colony size on plates with multiple colonies, Chacón, et al. [5] assumed that interactions from neighboring colonies are additive, resulting in interaction terms defined by $\sum\frac{1}{D}$ and $\sum\frac{1}{D^2}$. We implemented calculation of these metrics within ColTapp. We hypothesize that not only the location but also the size of colonies surrounding the focal colony is likely to play a role in the interaction magnitude. Thus, we calculate an additional metric: the sum of angular diameters, calculated as $AD = \sum 2\arctan(\frac{R_i}{D_i})$, where R_i and D_i are the radius and

distance from the focal colony of each neighboring colony. This metric, unexplored in the context of colony growth, is biologically plausible since it assumes that distant large colonies (that have consumed large amount of resources) have a similar effect as small but closer colonies, consuming resources closer to the focal colony. A user may define distance cutoffs to limit the interaction terms to colonies within a maximal distance to the focal colony for further calculations.

Finally, Chacón, et al. [5] proposed that the amount of nutrients available to each colony on the agar plate can be approximated with Voronoi cell areas, obtained by tracing perpendicular bisector lines between each pair of neighboring colonies [6]. They showed that the final size colonies attain when agar plate carrying capacity is reached can be predicted by the colonies Voronoi cell areas. ColTapp computes these areas by performing a Voronoi tessellation within user-specified boundaries [7].

5 Tracking radius over time

5.1 Drift correction

Slight drift can occur in time-lapse setups during imaging. Therefore, ColTapp incorporates an image registration algorithm which performs a 2-D rigid translation at subpixels resolution (1/κ), where the factor κ (adjustable as *RegistrationFactor* in *Options*) can be specified by the user. The algorithm computes the up-sampled cross-correlation with a discrete Fourier transform [8]. The drift correction is done on a user-defined small area of the image by generating a translation vector in respect to the reference frame. Concretely, the translation is not applied to the images themselves, but to the colonies' centers position.

5.2 Colony centers correction

The centering of colony circles is crucial to track radii over time, especially at early timepoints when colonies are small. ColTapp offers an automatic center correction, by tentatively detecting circles on sub-images cropped from an early frame based on the location and radii of the colonies detected on the reference frame. These newly detected circles' center coordinates are kept as colony center, unless they are farther away than a user-defined threshold from the original coordinates or if they are set to the center coordinates of another colony in close proximity. In these cases, the correction is skipped, and the colonies are added to a list for subsequent manual center correction. If no circle is detected at all because the colony is not yet visible at that timepoint, the same process is repeated 10 frames later. The user is able to monitor this process visually.

Alternatively, the user may choose to execute this task manually, by clicking the correct center on the sub-images sequentially displayed.

5.3 Overlapping colonies

Neighboring colonies can be visible as blurry regions in the top of kymographs, hindering creation of clean binary images (Fig. 5B). To reduce this phenomenon, before the kymograph creation process, overlapping colonies are automatically detected and the ranges of angles corresponding to adjacent colonies are discarded from the polar transformed intensity data. If more than 90% of all angles are discarded because of overlap, the exclusion of angles is omitted completely, to avoid reducing the available data too much. The overlap detection functionality can be deactivated or tuned with *Scale radius for overlap* (accessible in *Options*). This scaling factor is multiplied to the radius of the focal colony from which center neighboring colonies are tested for overlap: by increasing it, a user may choose to discard ranges of angles corresponding not only to overlapping colonies but also very close colonies. Note that this increase might lead to high proportions of angles to be discarded. Decreasing the scaling factor leads to reduced ranges of excluded angles. This might be useful in densely populated plates to still achieve some overlap exclusion to increase quality of kymographs at earlier timepoints.

5.4 Radial growth curve correction

ColTapp has an inbuilt function to automatically detect radial growth curves with probable errors based on the number of local maxima, size of radius differences from frame to frame, monotonicity, and number of frames without a successful radius determination. These (or any) radial growth curves can be manually corrected with a dedicated tool (Fig. S3) which allows the user to switch for each colony individually between Global thresholding and Edge detection method, and adjust any parameter of the two methods to derive best parameter combinations to derive the radial growth curve from the kymograph.

5.5 Algorithm performance

Computational time was assessed on a subset of 10 time-lapse sequences (each of 410 or 423 frames) from our demonstration dataset. Total required time was proportional to the number of frames and colonies to process, because each frame and colonies are processed sequentially (Supplementary table S3). Therefore, computational time per colony and per frame is most representative of the computational efficiency of the colony radius tracking algorithm (average = 0.079 s, SD = 0.047 s). Additionally, the computational time was observed to increase with the size of the colonies on the *reference frame*, as the sub-images used for radius tracking are bigger.

We assessed the accuracy of the algorithm by manually evaluating the quality of the 1411 kymograph derived radial growth curves. The *Global thresholding* method usually yields correct binary images except for complex kymographs resulting from high amount of colony

overlap and/or lighting artifacts. When using the default *Global thresholding* method to detect the kymographs' edges, many growth curves of our dataset were classified as incorrect (mean = 21%, SD = 15%) (Supplementary table S3). ColTapp inbuilt automatic quality assessment function detected on average 83% (SD = 9%) of these incorrect growth curves (Supplementary table S3).

6 Reference growth parameters definition

The user can define the reference radial growth rate and appearance time either by providing known values manually, or by directing ColTapp to a folder containing a control experiment monitored by time-lapse and already analyzed, to extract these parameters automatically. In this case, ColTapp obtains the mean of appearance time and radial growth rates of the control experiment's colonies. Users concerned by the presence of outliers in the growth control experiment may change the mean to median (or any user-defined quantile) in the *Options*. These reference experiments can be used for further calibration.

7 Sequence of endpoint images

7.1 Delayed colony detection

Some very late appearing colonies may not be observed at the optimal time for colony size observation (e.g. 24h). In that case, we suggest capturing image(s) at later timepoint(s) (e.g. 48h). ColTapp allows the user to overlay images taken from the same plate at different times to ease detection of very late growing colonies which did not yet appear at an earlier timepoint. ColTapp assumes that a folder under analysis contains multiple images from the same timepoint and requires the user to navigate to a folder containing the same set of images with matching order, acquired at another timepoint.

To achieve similar image orientation at both timepoints, the user may place a mark on the edge of the plate as reference. Users can manually align images which have not been captured in the exact same orientation by clicking at two matching positions on the images of the two timepoints. The inbuilt MATLAB function *fitgeotrans* is used to fit a geometric transformation to the pairs of points with a nonreflective similarity to estimate rotation and translation of the two images to align. If delayed colonies are revealed at this point, the user has the option to add not yet visible colonies to the earlier timepoint, which are defined as colonies with a radius equal to zero.

7.2 Sequence of endpoint images

The user can link corresponding folders of multiple image sets taken at different timepoints to create timeseries data without the need for a time-lapse imaging setup. Colonies on the images

in different folders are matched based on the minimal distance of centers between colonies. Mismatches can be corrected by aligning the images at different timepoints by fitting a geometric transformation to user-defined pairs of points with a nonreflective similarity to estimate rotation and translation of the two images to align. It is necessary that the number of detected colonies is the same in all folders.

8 Options

Many parameters of implemented functions can be tuned by the user within the *Options* window (Fig S2). The *Options* window is divided in four tabs (*Global*, *Detect*, *Main* (*TL* or *EP*) and *Visualize*). We describe some of the most important parameters and functions here.

The *Global* tab allows to set the method for converting a colored image into a grayscale version, activate direct data visualization options on the image (e.g. colony circles and Voronoi edges), set averaging strategy for reference growth data extraction and to define the unit (pixel or micrometer) of the data export function.

The *Detect* tab contains options to set image processing method (bright colonies on dark background, inversion or user-defined), choose colony detection method (Regionprops based circle detection or direct circle detection) and modify any of the parameters of the colony detection algorithm.

The *Main-TL* tab allows to tune parameters related to radial growth curve creation and appearance time determination. For example, definition of the default kymograph processing method, time interval between frames, *RegistrationFactor*, scaling of radius for overlap exclusion as well as R_{thresh} and Fr_{lin} are possible. Additional functions are subset radii tracking, manual timepoint specific radius correction, curve removal and interactive scaling of the radii defining the size of img_{crop}.

The *Main-EP* tab contains small functions to assign the AOI and spatial calibration factor of the current frame to all frames of the loaded folder and remove the linked folders.

Finally, the *Visualize* tab allows to tune details of the data previewing functions. Smoothing factor for radial growth curves, units to use for plotting (pixel or micrometer and frame or hour), number of bins used for distributions and titles can be set here.

9 Data export

The generated data is automatically saved in the same folder as the images are stored in the native MATLAB format (.mat files) when running the application. For export to another software, ColTapp provides a data export functionality. Through the export menu, user can save comma

separated values files of position, radius, fitted appearance time, as well as several calculated density measures and simple morphology descriptors of each colony.

Most variables are exported as a three-column table, containing the frame number, the colony number and the exported data of interest, grouped with similar variables. In TL mode, the radius of colonies is exported as a matrix of time by colony to facilitate extraction of growth curves.

The user can choose to export relevant variables either as pixel values or as µm values, provided the spatial calibration factor was set on all frames. Additional data as for example the spatial calibration factor, used color to grayscale transformation method as well as plate center and radius (if specified) can be exported in a separate metadata csv file.

Figures

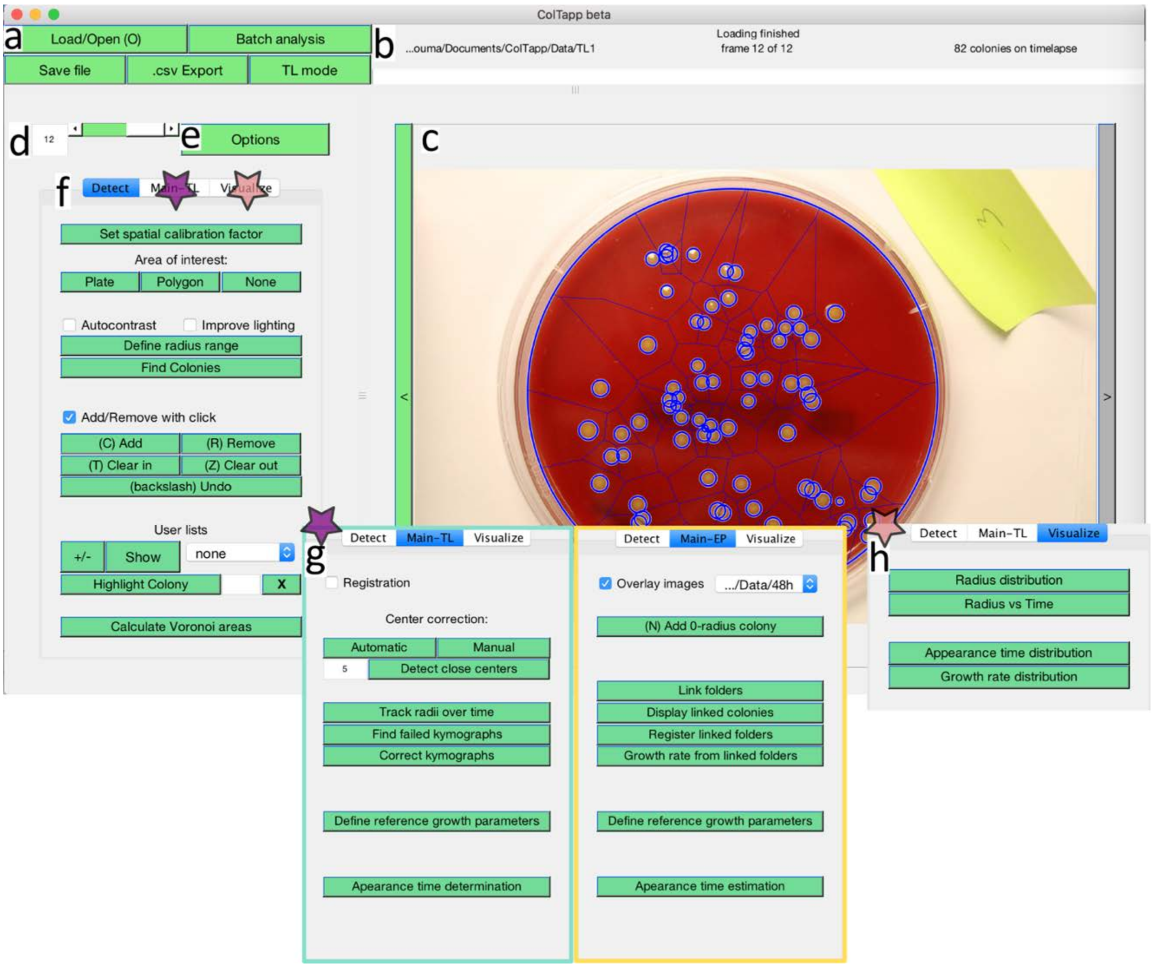

Supplementary Figure 1: The ColTapp graphical user interface, described in SI 1. Note that the color stars on the graphical user interface show the access to the 2 other tabs following the *Detect* tab. The display of the corresponding tabs is shown below. Note that the *Main* panel will be either the left version (contoured in turquoise) or right version (contoured in yellow) depending on the mode the user chose.

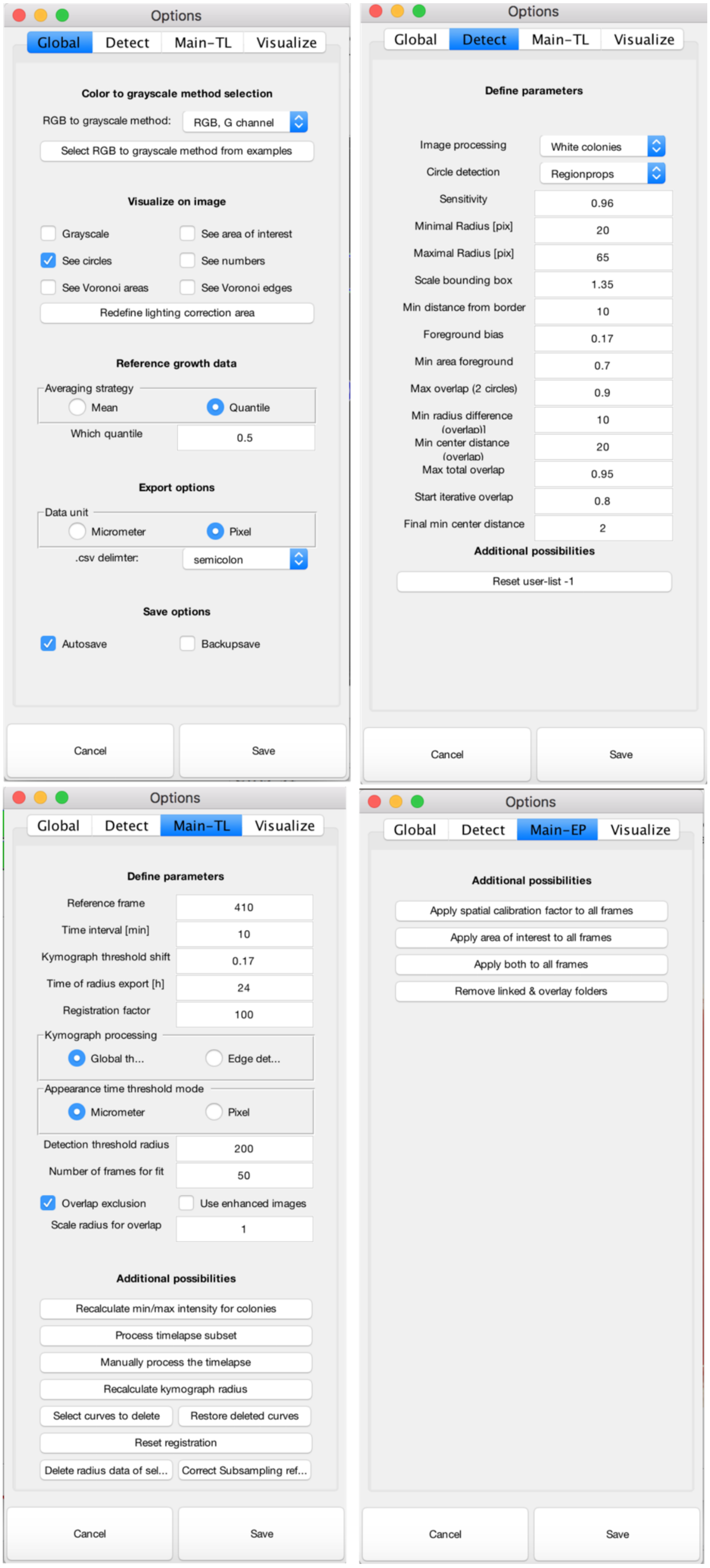

Supplementary Figure 2: The ColTapp options window, described in Supplementary text 9.

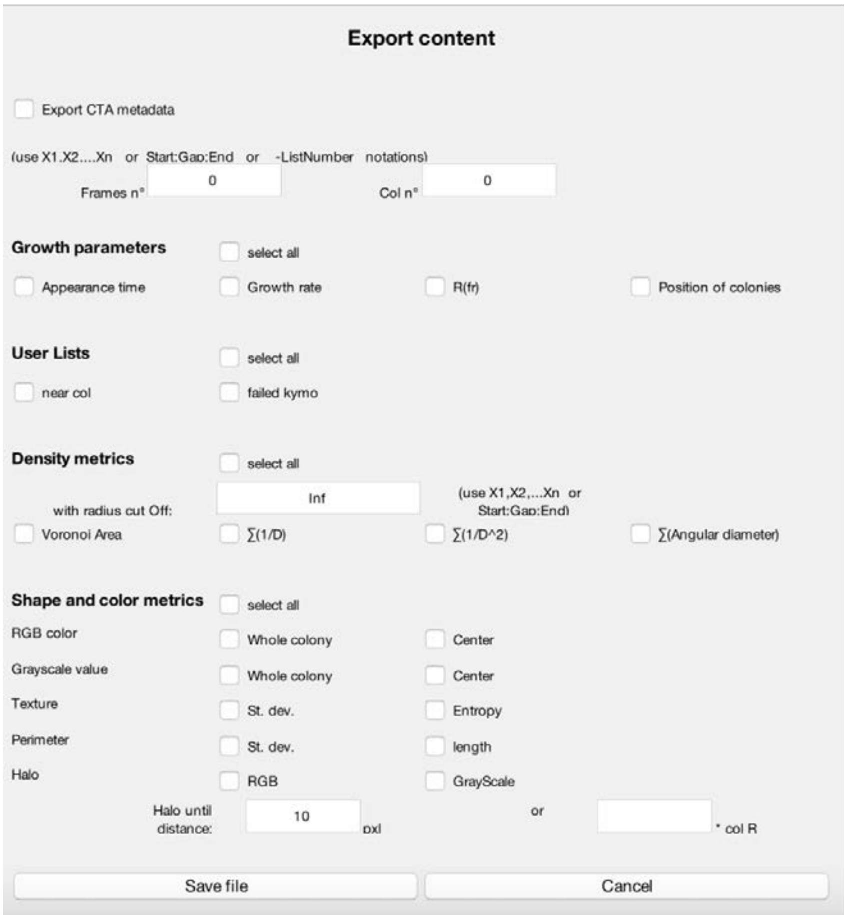

Supplementary Figure 3: The ColTapp export window. The export functionality is described in Supplementary text 10.

a

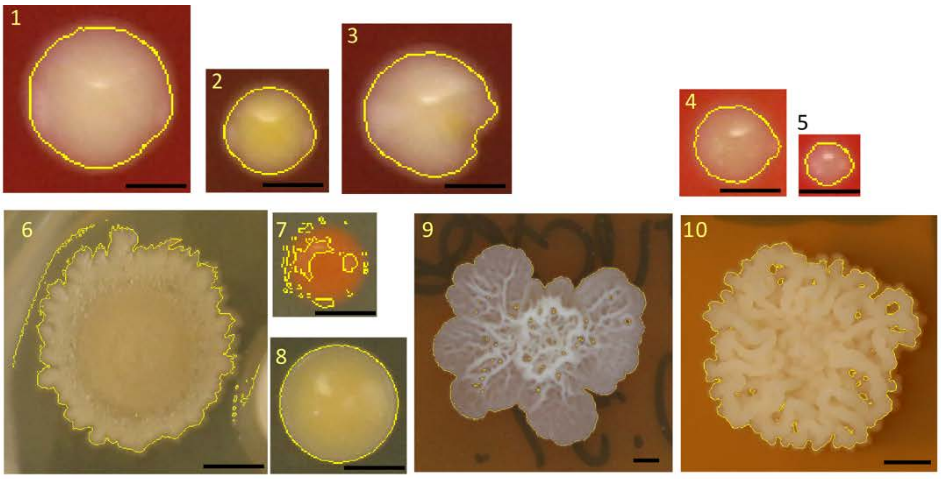

b

	1 *S.aureus*	2 *S.aureus*	3 *S.aureus*	4 *S.aureus*	5 *S.aureus*	6 Unknown	7 Unknown	8 Unknown	9 *S. cervisiae*	10 *S. cervisiae*
Radiusum	1167	929	1162	855	439	5155	823	1592	6441	3383
MeanR	184	167	171	197	199	158	165	175	135	179
MeanG	125	120	122	117	99	133	99	150	114	137
MeanB	98	67	93	85	81	82	42	84	89	83
CntrR	218	210	219	205	206	178	191	198	146	192
CntrG	182	173	185	151	133	146	93	166	130	156
CntrB	137	67	138	106	111	86	21	80	100	101
HaloR	131	99	104	193	194	117	106	95	92	146
HaloG	32	39	32	60	41	102	98	86	62	79
HaloB	20	22	20	31	28	62	66	54	27	12
MeanGray	0.45	0.43	0.44	0.46	0.38	0.48	0.33	0.56	0.40	0.46
CntrGray	0.69	0.66	0.71	0.59	0.52	0.53	0.31	0.63	0.47	0.55
HaloGrey	0.06	0.09	0.06	0.23	0.15	0.34	0.33	0.29	0.18	0.20
TxturStd	0.18	0.18	0.19	0.10	0.11	0.09	0.02	0.06	0.16	0.14
TxturEnt	7.20	7.17	7.29	6.56	6.50	5.96	4.10	5.68	6.60	6.29
PerimStd	1	1	2	1	1	16	6	1	123	25
PerimLength	186	141	187	135	63	2019	268	295	7736	2262
AlphaRemov	0	0	0	0	0	0	0	0	0	0

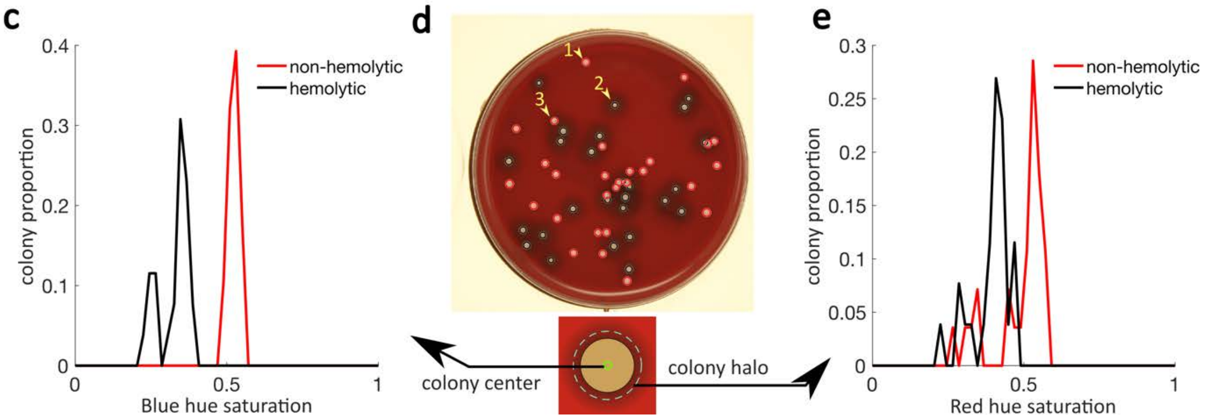

Supplementary Figure 4: Morphology descriptors. (**a**) A selection of colonies from different species growing on different media: 1-5 *S. aureus* clinical isolates on CSB; 6-8 Unknown contaminants on YPD (0.25% glucose); 9-10 *S. cervisiae* on YPD (0.25% glucose). The scale shown on each image corresponds to 1000 µm. The yellow pixels represent the perimeter edge derived by ColTapp for each colony. Note that it failed for colony 7, which is darker than its background. Moreover, note that when the background or the colony surface is inhomogeneous (e.g colony 6 and colony 10 respectively) false positive "edges" are detected. (**b**) ColTapp morphology and color output data. The radius size of the detected colony circle is shown in first row (in micrometers). RGB color values and grayscale color values follow for either the whole surface in the detected colony circle, the colony center (within a 5 pixels radius) or the colony halo are shown. Note that cells are colored to reflect the values they

display. Then, simple colony morphology descriptors: texture (calculated either as standard deviation of the color or entropy within the whole surface in the detected colony circle), perimeter length (number of pixels detected as "edge", visualized in yellow in (**a**) or perimeter standard deviation (std of the distance from the colony center of each of these pixels). The last readout (AlphaRemov) corresponds to the angle excluded for the calculation of these parameters in case of adjacent colony. (**c-e**) An example of the usage of these readouts (**d**) Two different *S. aureus* strains (a non-hemolytic one VS. a hemolytic and yellowish one) formed colonies on a CSB plate and can be differentiated with ColTapp. The more yellow colonies present less blue saturation (**c**, histogram of 50 bins in a 255 scale). They also appear with a dark halo due to hemolysis of the blood contained in the agar, which results in lower red saturation in the colony halo (**e**, histogram of 50 bins in a 255 scale). Note that colonies pointed with yellow arrays in d are the colonies 1-3 shown in (**a**) and analysed in (**b**). Colony 3 is a colony from the non-hemolytic strain but note that its proximity to colonies of the hemolytic strain results in a darker halo for it as well.

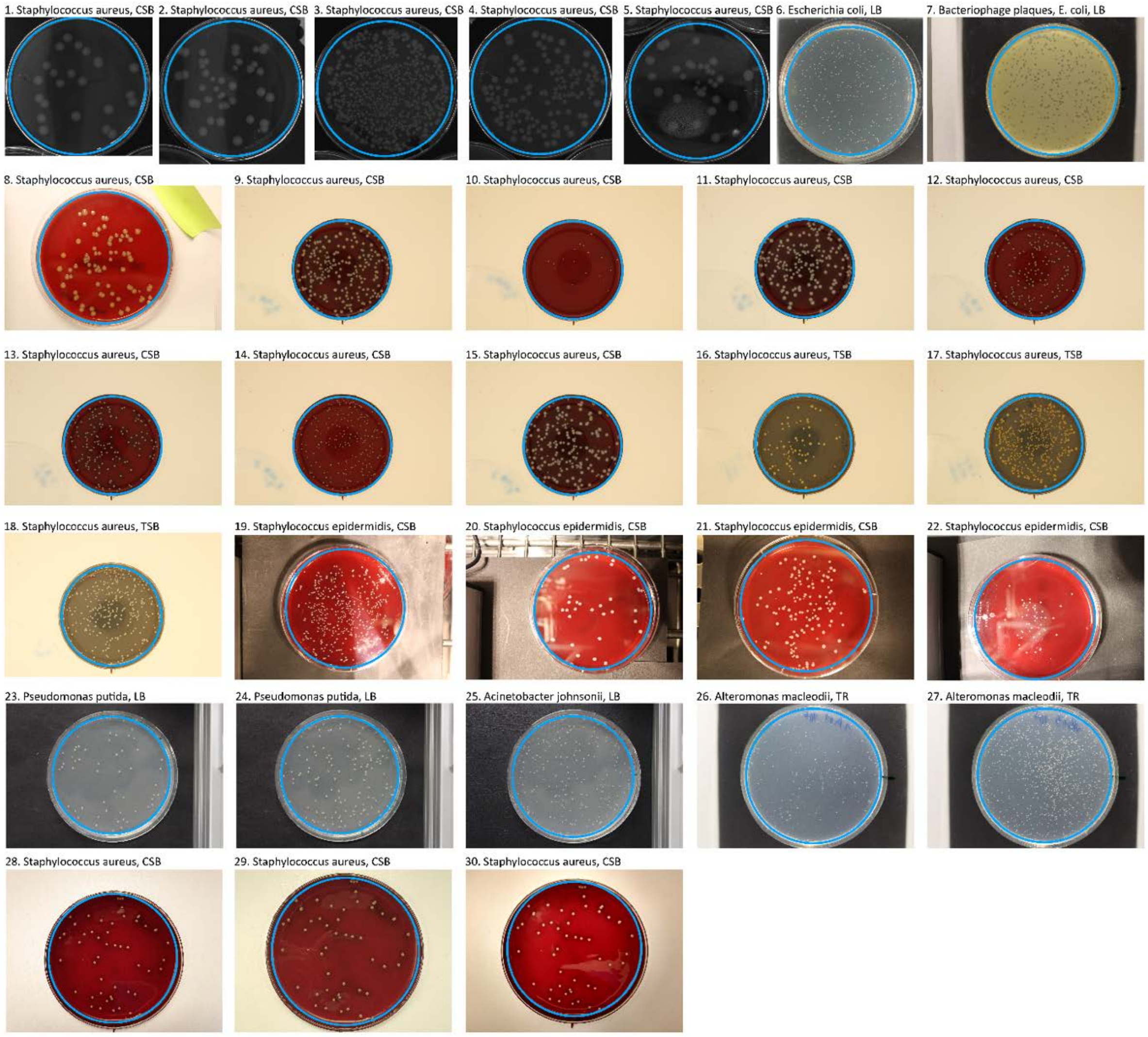

Supplementary Figure 5: The 30 images used for assessment of computational time and accuracy of the colony detection algorithm (Strains and growth conditions in Supplementary table S1, results in Supplementary table S2). The area of interest was set to the displayed blue circle on each image. Utilized cameras: images 1-5: Basler acA5472-17um 20MP with Fujinon Objective CF16ZA-1S 16mm/1.8M37.5x0.5; images 7-27: Canon EOS 1200D; image 28 and 29: Samsung Galaxy Note 10 (28: 12MP wide-angle lens, 29: 12MP Telephoto lens); image 30: Motorola Moto G5. CSB: Columbia sheep blood, LB: lysogeny broth, TSB: tryptic soy broth, TR: Tibbles Rawling, 0.5% sucrose.

Supplementary Figure 6: Accuracy of colony detection. A collection of 26 images of different species and agar media were used to benchmark the colony detection accuracy. (**a**) False positive rate was not increasing with total number of colonies on a given image (Pearson correlation, p = 0.091). Some images had imperfect lighting conditions which resulted in glares and reflections. Many false positive circles were detected in clusters in these regions (blue circles, fp: false positive). (**b**) False negative rate was correlated with total number of colonies (Pearson correlation, p = 0.00037). We did not observe an association between false negative rate and false positive clusters.

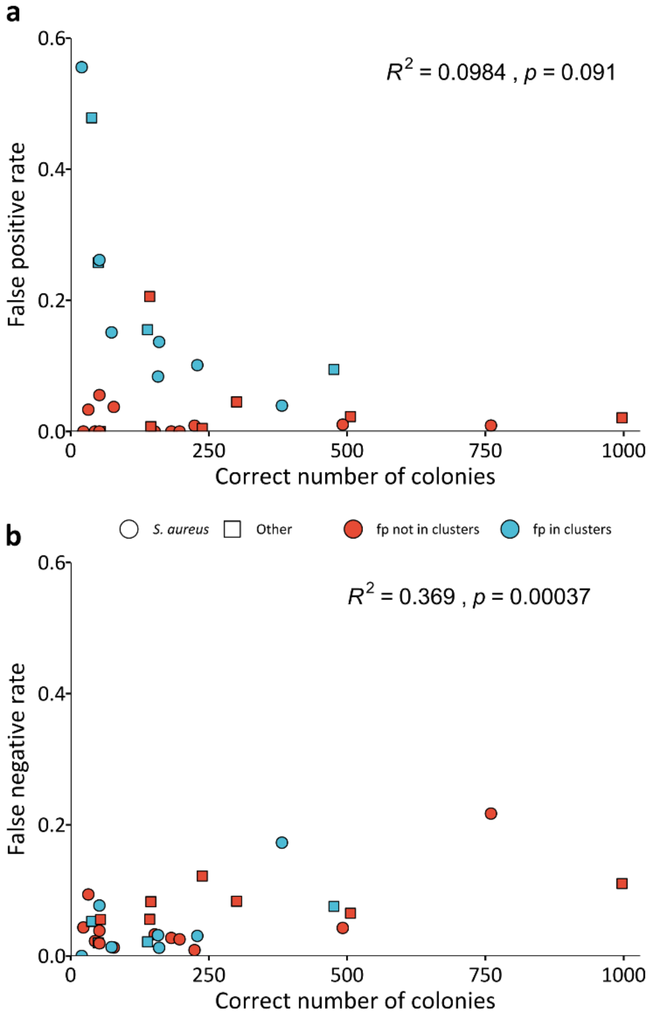

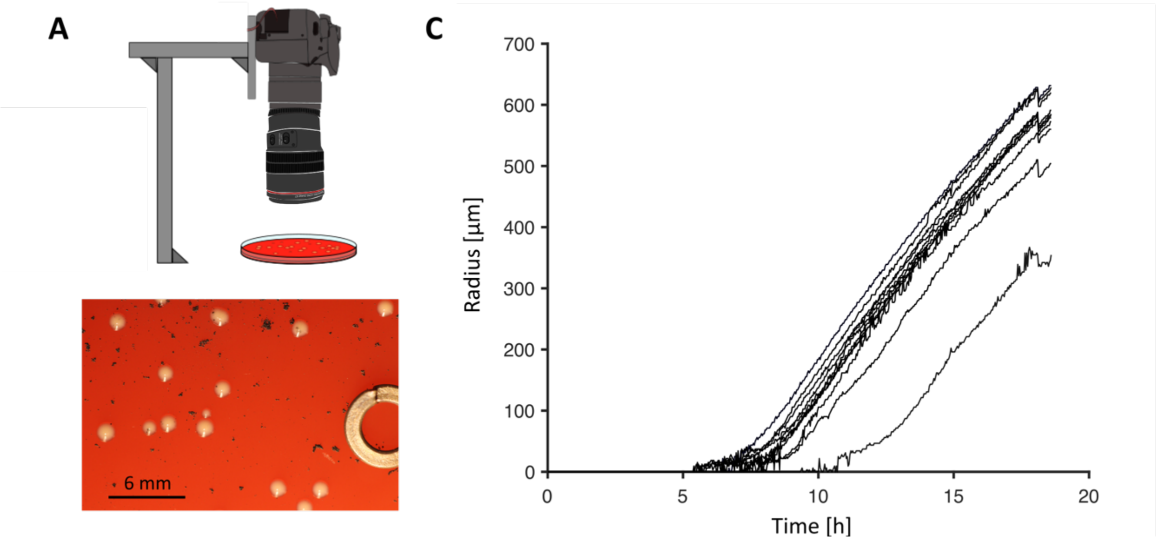

Supplementary Figure 7: Close up colony growth. (**a**) The zoom factor of the images can easily be increased to a level were the exponential phase of bacterial colony growth can be observed, by mounting commercially available macro lenses to a camera. (**b**) As an example, S. aureus bacterial colonies are grown on a blood plate. Some particles of activated charcoal are visible on the plate to help with focusing when experiment is setup, and a metal ring on the right of the image serves as a distance reference. (**c**) With this setup, the distance resolution can be brought below 50µm, and a short exponential phase is observed when colonies grow, before they reach ~100 µm. In this case, the appearance time cannot be estimated using a simple linear regression and calculations will need to be adapted.

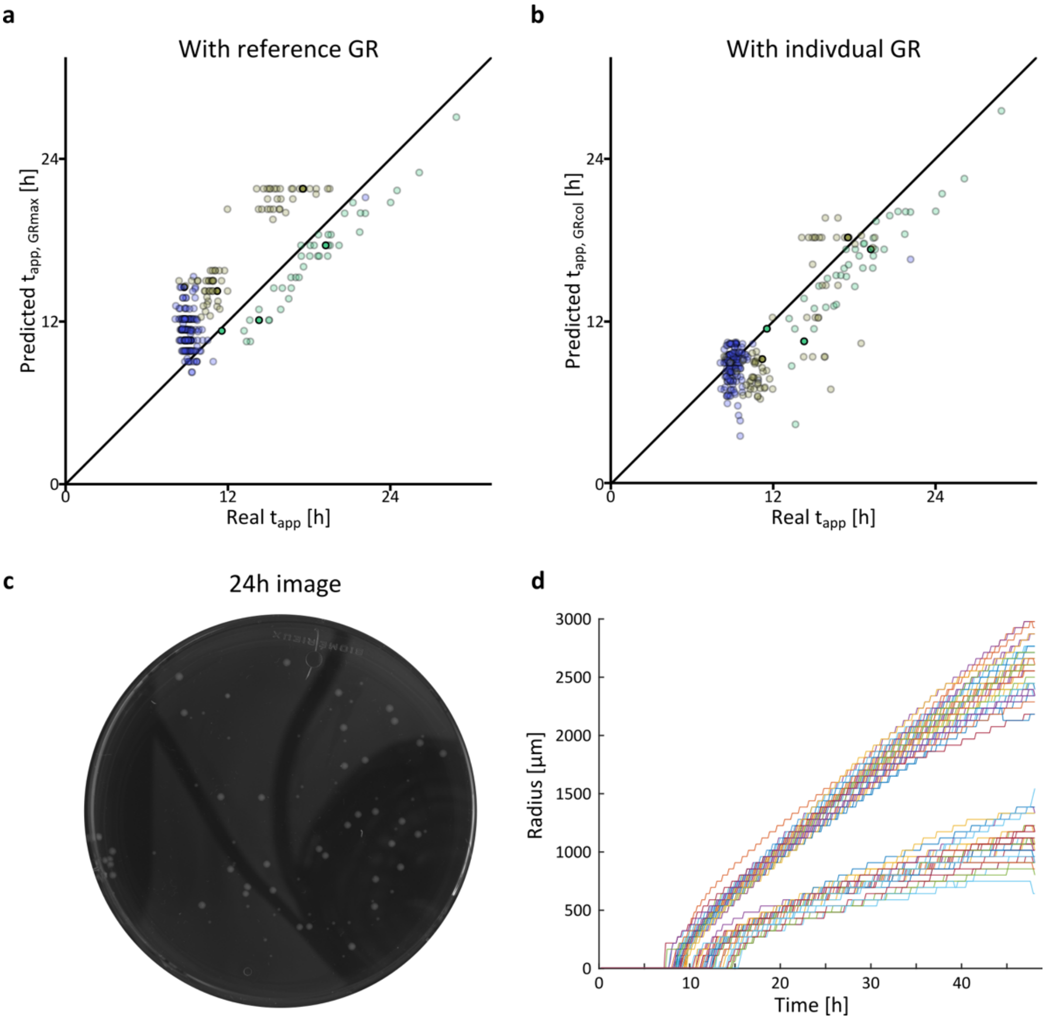

Supplementary Figure 8: **(a, b)** Correlation plots of the linear regression-based estimation of appearance time (t_{app}) either with **(a)** a reference growth rate (GR_{max}) of 65 µm/h or **(b)** growth rate estimated for each colony (Gr_{col}). The real t_{app} was determined from time-lapse analysis. **(c)** The image of the time-lapse at 24h. **(d)** Growth curves obtained upon time-lapse analysis.

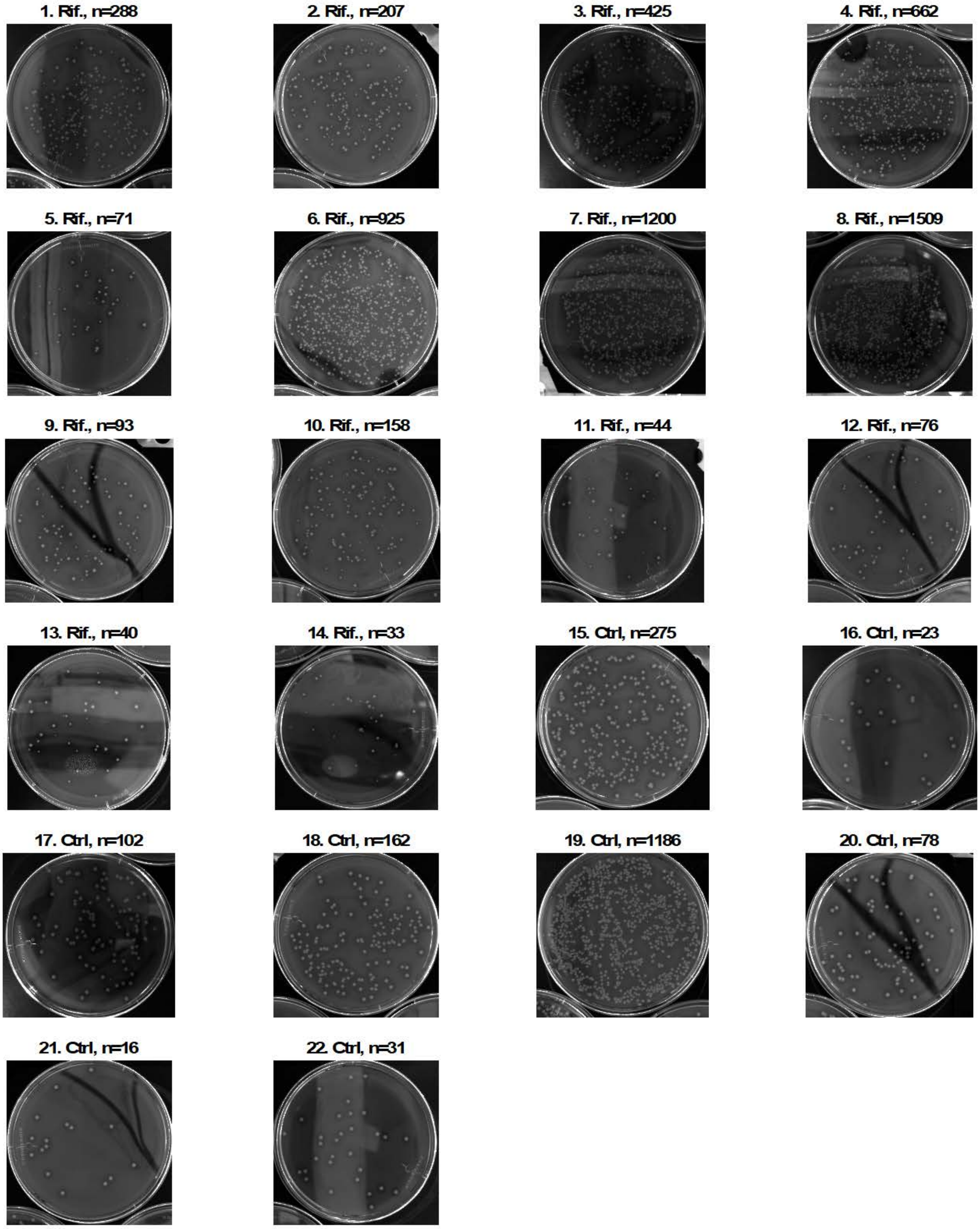

Supplementary Figure 9: Images at 24h of the 22 plates used for the training (Ctrl, exponential growth phase) and testing (Rif, rifampicin pre-treated) dataset.

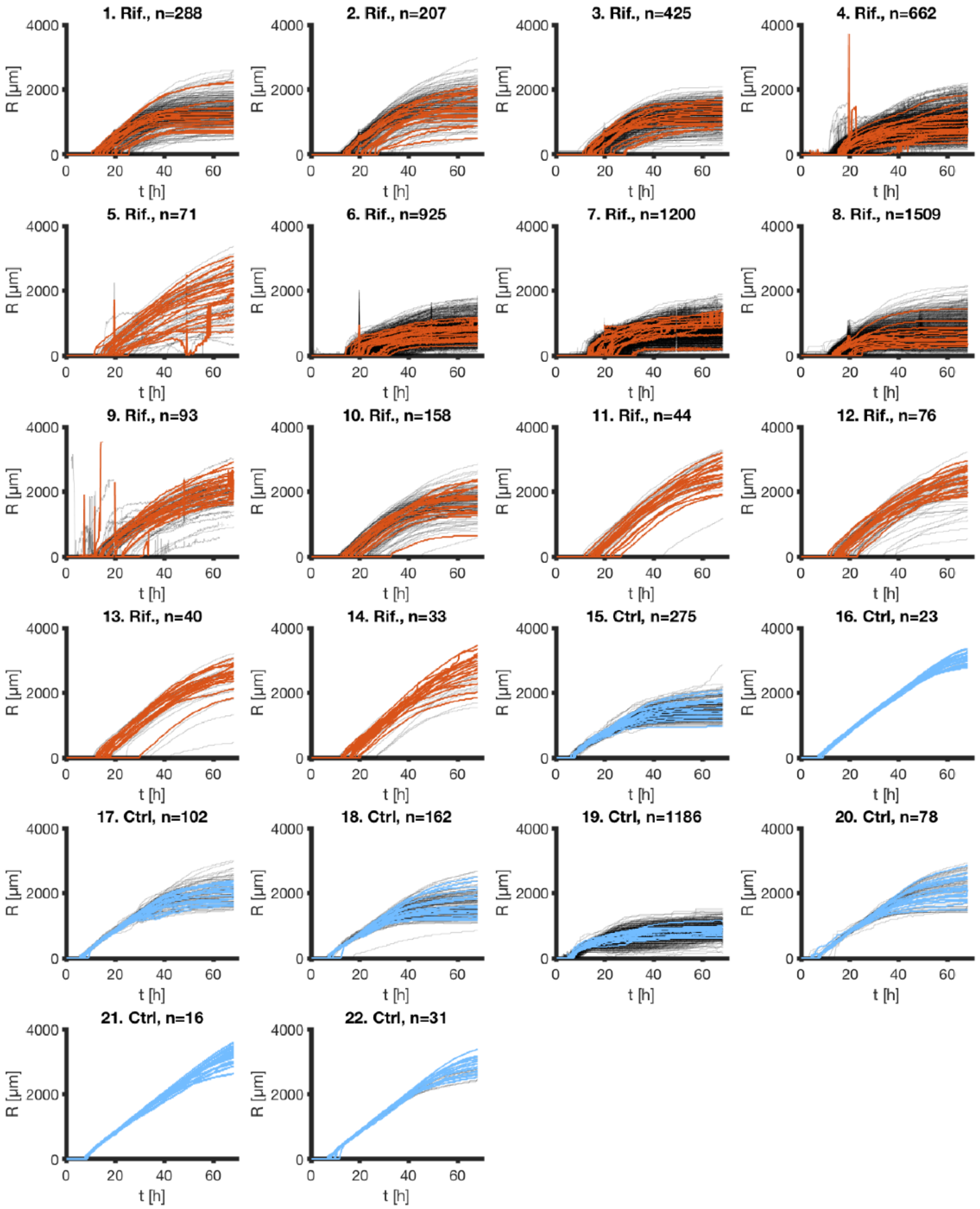

Supplementary Figure 10: Growth curves obtained for the training (blue) and testing (orange) data set. All curves are displayed in black and 20 random growth curves are colored for each plate to illustrate the general dynamics.

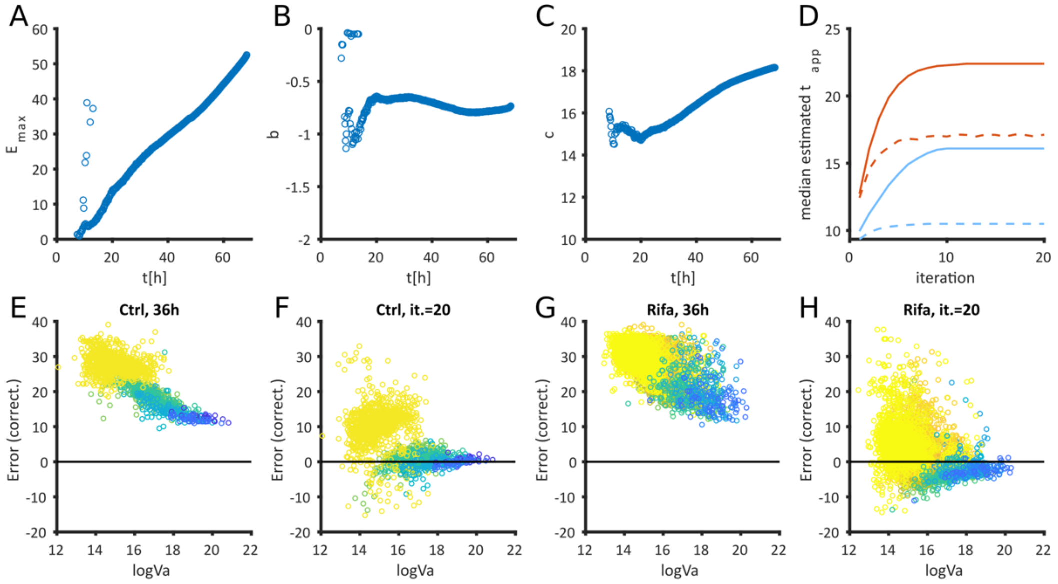

Supplementary Figure 11 Panels (**a**), (**b**) and (**c**) show the values of the parameters obtained by fitting the training data (panel E) at different data points (see also SI movie 3). The prediction of the parameters is not very noisy and consistent, with the dominating change being the value of the maximal error. (**d**) Representation of the median predicted tapp on each iteration of the correction shows that the predictions stabilize in fewer than 10 iterations. Panel (**e**) and (**g**) show the uncorrected error of plates at late time point (36h) for control and rifampicin dataset, respectively. Panel (**f**) and (**h**) are the corrections obtained after 20 iterations.

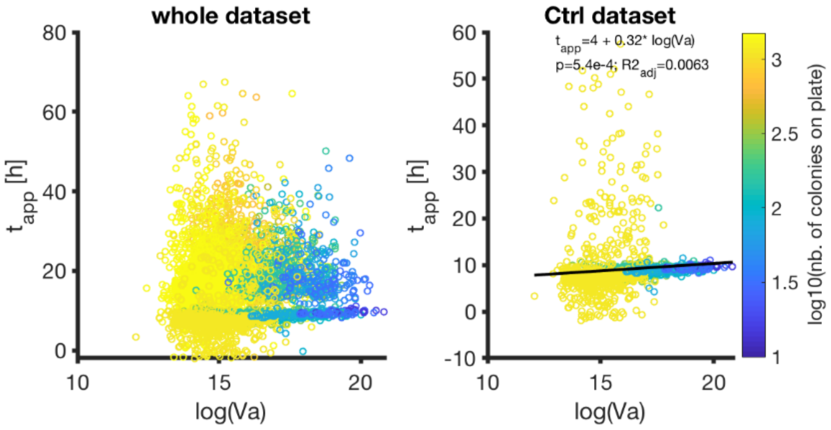

Supplementary Figure 12: Visualization of the effect of local density (log(Va) on the appearing time, either on the whole dataset (**left**) or solely on the control dataset (**right**). We didn't notice an important effect of density on t_{app}, and we attribute the observed slope to noise in the very dense plate data.

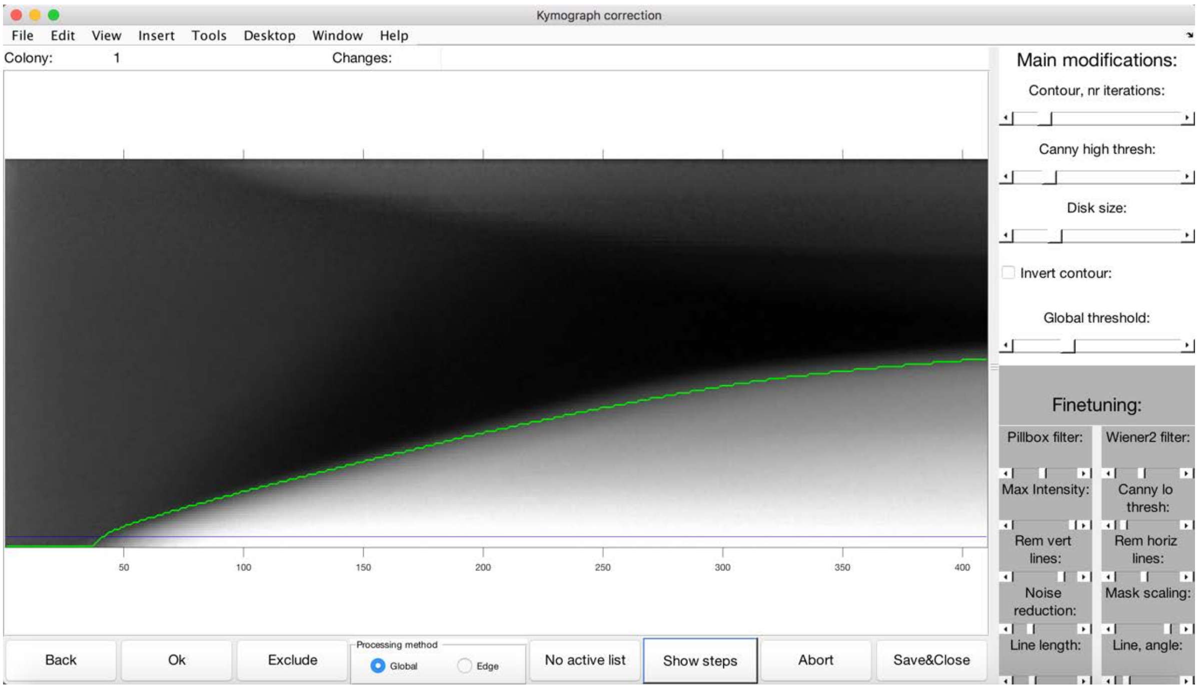

Supplementary Figure 13: The ColTapp kymograph correction window. The user can visualize kymographs and the derived radial growth curve (shown here in green) sequentially and correct the growth curve manually. If the Global method does not perform well, a user may choose to change to the Edge detection method. All parameters for both methods are tweakable with the sliders in the panel on the right side. Additionally, buttons to exclude the currently displayed colony, adding it to a list and display all steps of the Edge detection method in a popup window for better determination of the source of error are available.

Tables

Image Nr.	Species	Strain	Agar medium	Incubation temp (°C)	Picture timepoint
1	*S.aureus*	JE2	CSB	37	70h
2	*S.aureus*	JE2	CSB	37	70h
3	*S.aureus*	JE2	CSB	37	70h
4	*S.aureus*	JE2	CSB	37	70h
5	*S.aureus*	JE2	CSB	37	70h
6	*E.coli*	MG1565	LB	37	48h
7	Phage plaques	Qβ	LB, *E.coli*	37	24h
8	*S.aureus*	Cowan I	CSB	37	48h
9	*S.aureus*	Clin. Iso.	CSB	37	48h
10	*S.aureus*	Clin. Iso.	CSB	37	24h
11	*S.aureus*	Clin. Iso.	CSB	37	48h
12	*S.aureus*	Clin. Iso.	CSB	37	24h
13	*S.aureus*	Clin. Iso.	CSB	37	48h
14	*S.aureus*	Clin. Iso.	CSB	37	24h
15	*S.aureus*	Clin. Iso.	CSB	37	48h
16	*S.aureus*	Clin. Iso.	TSB	37	48h
17	*S.aureus*	Clin. Iso.	TSB	37	48h
18	*S.aureus*	Clin. Iso.	TSB	37	48h
19	*S.epidermidis*	ATCC12228	CSB	37	24h
20	*S.epidermidis*	ATCC12228	CSB	37	24h
21	*S.epidermidis*	ATCC12228	CSB	37	24h
22	*S.epidermidis*	ATCC12228	CSB	37	24h
23	*P.putida*	KT2440	LB	30	24h
24	*P.putida*	KT2440	LB	30	24h
25	*A.johnsonii*	C6	LB	30	24h
26	*A.macleodii*	Env. Iso.	TR	25	48h
27	*A.macleodii*	Env. Iso.	TR	25	48h
28	*S.aureus*	CI	CSB	37	24h
29	*S.aureus*	CI	CSB	37	24h
30	*S.aureus*	CI	CSB	37	24h

Supplementary Table 1 Colony detection benchmark images. We tested the accuracy and computational efficiency of the colony detection algorithm with a set of 30 images (Supplementary Fig S5, S6). This table lists the species, strain, agar medium, incubation temperature and incubation time (picture timepoint) for each plate. Images Nr. 1 to 5 correspond to some examples of the main dataset, also visualized in Supplementary Fig. S9 and S10. Clin. Iso: clinical isolate; Env. Iso: environmental isolate; CSB: Columbia sheep blood; LB: lysogeny broth; TSB: tryptic soy broth; TR: Tibbles Rawling, 0.5% sucrose; PBS: phosphate buffered saline.

Image Nr.	Color space	Species	Total time [s]	Time per circle [s]	N initial circles	N false positive	N false negative	N corrected	False positive rate [%]	False negative rate [%]	False positive in clusters	Average radius [pixel]	SD radius [pixel]	Min. expected radius [pixel]	Max. expected radius [pixel]	Open CFU initial N	Open CFU init/ correct	ColTapp init/ corect
1	Gray	*S.aureus*	2.22	0.1009	22	0	1	23	0.00	4.35	no	62.91	3.94	45	77	0	0.00	0.96
2	Gray	*S.aureus*	2.38	0.0553	43	0	1	44	0.00	2.27	no	52.66	9.03	36	64	11	0.25	0.98
3	Gray	*S.aureus*	9.59	0.0125	767	7	165	760	0.91	21.71	no	17.87	5.94	8	39	342	0.45	1.01
4	Gray	*S.aureus*	3.51	0.0239	147	0	5	152	0.00	3.29	no	34.53	7.54	22	63	33	0.22	0.97
5	Gray	*S.aureus*	2.97	0.0990	30	1	3	32	3.33	9.38	no	54.53	11.28	26	89	8	0.25	0.94
6	RGB	*E.coli*	6.40	0.0222	288	13	25	300	4.51	8.33	no	7.00	0.85	6	16	282	0.94	0.96
7	RGB	*Phage plaques*	12.76	0.0264	484	11	33	506	2.27	6.52	no	18.66	6.61	8	35	332	0.66	0.96
8	RGB	*S.aureus*	9.20	0.1150	80	3	1	78	3.75	1.28	no	54.10	8.76	35	76	54	0.69	1.03
9	RGB	*S.aureus*	6.86	0.0306	224	2	2	224	0.89	0.89	no	33.23	5.13	10	27	174	0.78	1.00
10	RGB	*S.aureus*	3.70	0.0822	45	25	0	20	55.56	0.00	yes	14.15	3.42	6	29	20	1.00	2.25
11	RGB	*S.aureus*	7.16	0.0405	177	0	5	182	0.00	2.75	no	38.59	5.42	26	69	136	0.75	0.97
12	RGB	*S.aureus*	4.94	0.0270	183	25	2	160	13.66	1.25	yes	20.72	2.54	9	31	153	0.96	1.14
13	RGB	*S.aureus*	4.86	0.0291	167	14	5	158	8.38	3.16	yes	20.02	2.25	11	32	155	0.98	1.06
14	RGB	*S.aureus*	5.35	0.0217	247	25	7	229	10.12	3.06	yes	14.58	3.17	7	32	216	0.94	1.08
15	RGB	*S.aureus*	7.60	0.0396	192	0	5	197	0.00	2.54	no	35.43	7.03	14	53	156	0.79	0.97
16	RGB	*S.aureus*	3.91	0.0455	86	13	1	74	15.12	1.35	yes	25.21	6.17	8	46	72	0.97	1.16
17	RGB	*S.aureus*	7.67	0.0161	476	5	21	492	1.05	4.27	no	18.56	4.86	8	38	388	0.79	0.97
18	RGB	*S.aureus*	6.60	0.0201	329	13	66	382	3.95	17.28	yes	17.73	3.11	8	27	292	0.76	0.86
19	RGB	*S.epidermidis*	1.94	0.0040	486	46	36	476	9.47	7.56	yes	16.46	2.74	12	31	340	0.71	1.02
20	RGB	*S.epidermidis*	6.81	0.0987	69	33	2	38	47.83	5.26	yes	42.10	2.70	53	56	36	0.95	1.82
21	RGB	*S.epidermidis*	9.34	0.0580	161	25	3	139	15.53	2.16	yes	31.79	7.62	11	57	124	0.89	1.16
22	RGB	*S.epidermidis*	5.97	0.0905	66	17	1	50	25.76	2.00	yes	21.37	1.55	17	36	49	0.98	1.32
23	RGB	*P.putida*	5.27	0.1033	51	0	3	54	0.00	5.56	no	21.62	2.06	19	33	42	0.78	0.94
24	RGB	*P.putida*	6.26	0.0467	134	1	12	145	0.75	8.28	no	21.42	1.76	16	33	130	0.90	0.92
25	RGB	*A.johnsonii*	7.08	0.0337	210	1	29	238	0.48	12.18	no	15.16	1.12	12	23	207	0.87	0.88
26	RGB	*A.macleodii*	7.04	0.0414	170	35	8	143	20.59	5.59	no	14.28	2.49	12	25	100	0.70	1.19
27	RGB	*A.macleodii*	16.84	0.0186	906	19	110	997	2.10	11.03	no	12.33	1.29	11	22	788	0.79	0.91
28	RGB	*S.aureus*	5.16	0.0956	54	3	1	52	5.56	1.92	no	23.73	1.16	17	34	49	0.94	1.04
29	RGB	*S.aureus*	5.92	0.1184	50	0	2	52	0.00	3.85	no	28.02	1.64	23	39	49	0.94	0.96
30	RGB	*S.aureus*	5.11	0.0786	65	17	4	52	26.15	7.69	yes	26.10	1.50	23	39	46	0.88	1.25
		**Average:**	6.35	0.0532	213.63	11.80	18.63	214.97	9.26	5.56		27.16	4.16	17.30	42.37	159.47	0.75	1.09
		**SD:**	3.10	0.0351	215.58	12.61	36.19	230.59	13.89	4.94		14.20	2.80	11.86	18.51	165.12	0.26	0.28
		**Median:**	6.12	0.0409	164	9	4.5	148.5	3.54	4.06		21.52	3.14	12	35.5	127	0.79	0.99

Supplementary Table 2(see caption below)

Plate ID	N colonies	N frame	Total time [s]	Time per colony and frame [s]	Averag e radius [pixel]	N detected as failed	N false negative	N true negative	False negative rate [%]	N tn, need manual correction	N false positive	False detection rate [%]	N fp, need manual correction	N total false	Rate of failed, global [%]	N total need manual correction	Rate of manual correction needed [%]
1	288	423	4049.44	0.0332	27.7263	50	3	47	6.00	16	9	84.75	1	56	19.44	17	5.90
2	207	423	3403.52	0.0389	31.2876	30	5	25	16.67	6	8	78.95	2	33	15.94	8	3.86
3	425	423	8248.11	0.0459	35.0090	119	2	117	1.68	23	14	89.47	1	131	30.82	24	5.65
11	44	423	1517.18	0.0815	51.4680	2	1	1	50.00	0	1	66.67	0	2	4.55	0	0.00
12	76	423	1938.10	0.0603	42.7522	12	3	9	25.00	3	2	85.71	2	11	14.47	5	6.58
13	40	423	1596.20	0.0943	58.1285	7	1	6	14.29	0	1	87.50	0	7	17.50	0	0.00
14	33	423	1181.89	0.0847	54.8332	4	0	4	0.00	1	0	100.00	0	4	12.12	1	3.03
15	275	410	9261.21	0.0821	33.0484	124	0	124	0.00	6	29	81.05	1	153	55.64	7	2.55
16	23	410	1792.52	0.1901	56.9671	3	0	3	0.00	0	1	75.00	0	4	17.39	0	0.00
**Average:**	156.78	420	3665.35	0.079	43.47	39.00	1.67	37.33	12.63	6.11	7.22	83.23	0.78	44.56	20.88	6.89	3.06
**SD:**	146.53	5.73	3043.15	0.047	12.08	49.33	1.73	49.37	16.62	8.15	9.48	9.43	0.83	58.24	14.75	8.49	2.65
**Median:**	76.00	423	42.75	1938.10	0.08	12.00	1.00	9.00	3.00	2.00	1.00	11.00	5.00	84.75	17.39	3.03	6.00

Supplementary Table 3 (see caption below)

Supplementary Table 2: Colony detection benchmark. We tested the accuracy and computational efficiency of the colony detection algorithm with a set of 30 images (Supplementary Fig S5, S6). Detailed results for each image are displayed here. Average, standard deviation (SD) and median of each measurement are shown at the bottom of the table. We recorded number of detected circles by the algorithm, as well as the number of false positive of these and number of missed (false negative). We computed false negative and false positive rates with these numbers. Many of the images with high false positive rate had the false negative circles detected in large clusters, which is indicated on the table. Additionally, we measured computational time of the detection algorithm and report computational time per circle as well. Finally, average size of colonies in pixels and the range of expected radius is shown.

Supplementary Table 3: Radius tracking benchmark. We tested the accuracy and computational efficiency of the track radii algorithm with a subset of the dataset created for the density correction validation. Plate ID in the table are referring to ID numbers on Supplementary Fig. S9. Detailed results for each time-lapse are displayed here. Average, standard deviation (SD) and median of each measurement are shown at the bottom of the table. For testing, we set the default growth curve determination method to Global thresholding. We report here the number of colonies and frames of each time-lapse as well as the number of radial growth curves automatically identified as failed after applying the Global thresholding method, the number of growth curves falsely categorized as failed (including rate), and the number of the identified growth curves which needed more manual correction than a simple switch to the Edge detection method. Corresponding rates. Next, we manually assessed all growth curves not classified as failed and record number of falsely categorized as correct (including false detection rate). Of these manually identified, we assessed if more manual correction than a switch to Edge detection method was necessary. The combined number of automatically and manually identified growth curves is reported here and the rate of failure of the Global thresholding method is given. Overall, only few growth curves need manual correction after switching to the Edge detection method. Additionally, we report the total computational time and the time per colony and frame as these two factors are proportional to required time. Finally, Average colony size at the last frame of the time-lapse is indicated.

Movies

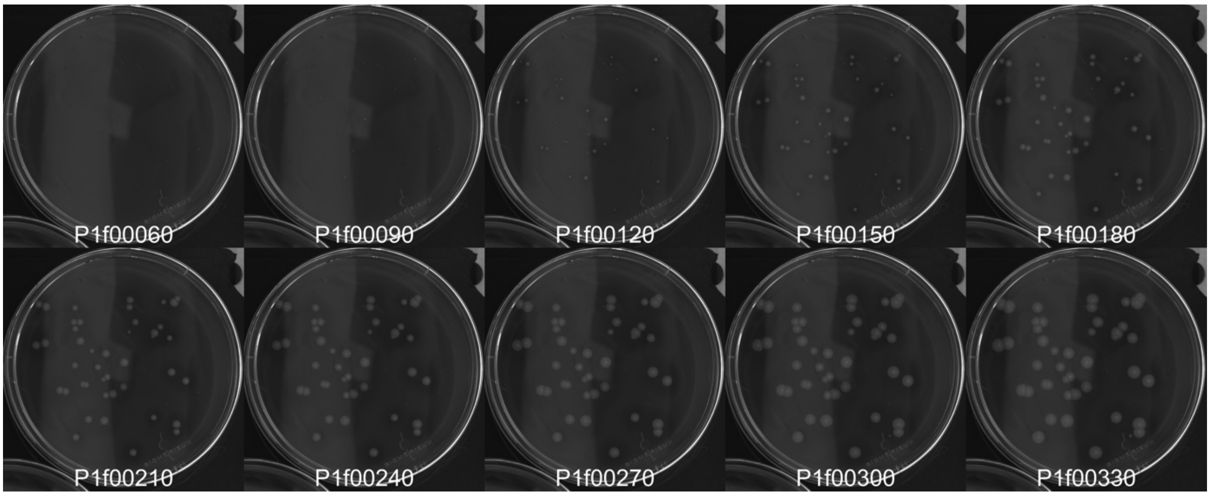

Supplementary Movie 1: Representative images of the SI movie 1 (one image every 5h, starting at 10h) that show a time-lapse of colony growth. The time-lapse movie is generated from the images of plate 11 (Fig. S9, Fig. S10). It includes 423 frames acquired in 10 min intervals.

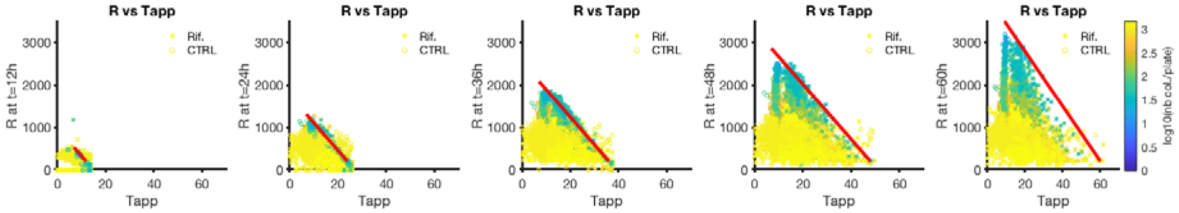

Supplementary Movie 2: This movie proposes to merge Fig 7BD (full dataset) and show the evolution of the correlation of radius of colonies and their appearance time. The red line represents the radius that could be obtained assuming the maximal growth rate, and deviations from this radius means that colonies grow slower than this maximal growth rate.

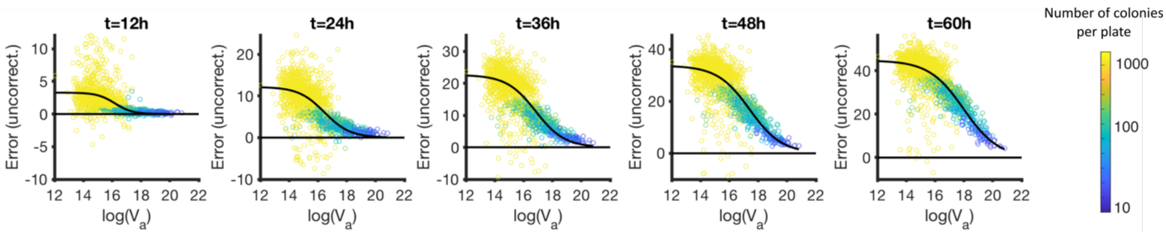

Supplementary Movie 3: This movie proposes to visualize as in Fig. 8A the correlation and fitted model of the systematic error obtained after the use of a linear regression with GR_{max} to obtain t_{app}. The 0 line thus represents colonies growing at maximal growth rate, and the fit is described in main text.

References

1 Pitol, A. K., Bischel, H. N., Kohn, T. & Julian, T. R. Virus Transfer at the Skin–Liquid Interface. *Environmental science & technology* **51**, 14417-14425 (2017).

2 Rodríguez-Verdugo, A., Vulin, C. & Ackermann, M. The rate of environmental fluctuations shapes ecological dynamics in a two-species microbial system. *Ecology letters* **22**, 838-846 (2019).

3 Simunovic, G. *et al.* Surface roughness assessing based on digital image features. *Advances in Production Engineering & Management* **11**, 93 (2016).

4 Palkova, Z. *et al.* Ammonia pulses and metabolic oscillations guide yeast colony development. *Molecular Biology of the Cell* **13**, 3901-3914, doi:10.1091/mbc.E01-12-0149 (2002).

5 Chacón, J. M., Möbius, W. & Harcombe, W. R. The spatial and metabolic basis of colony size variation. *The ISME Journal* **12**, 669-680, doi:10.1038/s41396-017-0038-0 (2018).

6 Okabe, A., Boots, B., Sugihara, K. & Chiu, S. N. *Spatial tessellations: concepts and applications of Voronoi diagrams*. Vol. 501 (John Wiley & Sons, 2009).

7 VoronoiLimit(varargin) v. 3.0.1.5 (Matlab Central File Exchange, Retrieved March 20, 2020, https://www.mathworks.com/matlabcentral/fileexchange/34428-voronoilimit-varargin, 2020).

8 Efficient subpixel image registration by cross-correlation (MATLAB Central File Exchange, Retrieved May 11, 2020, https://www.mathworks.com/matlabcentral/fileexchange/18401-efficient-subpixel-image-registration-by-cross-correlation, 2020).
